# Pinpointing novel risk loci for Lewy body dementia and the shared genetic etiology with Alzheimer’s disease and Parkinson’s disease: a large-scale multi-trait association analysis

**DOI:** 10.1186/s12916-022-02404-2

**Published:** 2022-06-22

**Authors:** Ping Guo, Weiming Gong, Yuanming Li, Lu Liu, Ran Yan, Yanjun Wang, Yanan Zhang, Zhongshang Yuan

**Affiliations:** 1 Department of Biostatistics, School of Public Health, Cheeloo College of Medicine, Shandong University, Jinan, 250012 Shandong China; 2 Institute for Medical Dataology, Cheeloo College of Medicine, Shandong University, Jinan, 250012 Shandong China; 3 School of Medicine, Cheeloo College of Medicine, Shandong University, Jinan, 250012 Shandong China

**Keywords:** Lewy body dementia, Alzheimer’s disease, Parkinson’s disease, Shared genetics, Multi-trait association analysis

## Abstract

**Background:**

The current genome-wide association study (GWAS) of Lewy body dementia (LBD) suffers from low power due to a limited sample size. In addition, the genetic determinants underlying LBD and the shared genetic etiology with Alzheimer’s disease (AD) and Parkinson’s disease (PD) remain poorly understood.

**Methods:**

Using the largest GWAS summary statistics of LBD to date (2591 cases and 4027 controls), late-onset AD (86,531 cases and 676,386 controls), and PD (33,674 cases and 449,056 controls), we comprehensively investigated the genetic basis of LBD and shared genetic etiology among LBD, AD, and PD. We first conducted genetic correlation analysis using linkage disequilibrium score regression (LDSC), followed by multi-trait analysis of GWAS (MTAG) and association analysis based on SubSETs (ASSET) to identify the trait-specific SNPs. We then performed SNP-level functional annotation to identify significant genomic risk loci paired with Bayesian fine-mapping and colocalization analysis to identify potential causal variants. Parallel gene-level analysis including GCTA-fastBAT and transcriptome-wide association analysis (TWAS) was implemented to explore novel LBD-associated genes, followed by pathway enrichment analysis to understand underlying biological mechanisms.

**Results:**

Pairwise LDSC analysis found positive genome-wide genetic correlations between LBD and AD (rg = 0.6603, se = 0.2001; *P* = 0.0010), between LBD and PD (rg = 0.6352, se = 0.1880; *P* = 0.0007), and between AD and PD (rg = 0.2136, se = 0.0860; *P* = 0.0130). We identified 13 significant loci for LBD, including 5 previously reported loci (1q22, 2q14.3, 4p16.3, 4q22.1, and 19q13.32) and 8 novel biologically plausible genetic associations (5q12.1, 5q33.3, 6p21.1, 8p23.1, 8p21.1, 16p11.2, 17p12, and 17q21.31), among which *APOC1* (19q13.32), *SNCA* (4q22.1), *TMEM175* (4p16.3), *CLU* (8p21.1), *MAPT* (17q21.31), and *FBXL19* (16p11.2) were also validated by gene-level analysis. Pathway enrichment analysis of 40 common genes identified by GCTA-fastBAT and TWAS implicated significant role of neurofibrillary tangle assembly (GO:1902988, adjusted *P* = 1.55 × 10^−2^).

**Conclusions:**

Our findings provide novel insights into the genetic determinants of LBD and the shared genetic etiology and biological mechanisms of LBD, AD, and PD, which could benefit the understanding of the co-pathology as well as the potential treatment of these diseases simultaneously.

**Supplementary Information:**

The online version contains supplementary material available at 10.1186/s12916-022-02404-2.

## Background

Lewy body dementia (LBD) is the second most common neurodegenerative dementia after Alzheimer’s disease (AD) [1, 2]. The population-based incidence of LBD reported in a large-scale systematic review is between 0.5 and 1.6 per 1000 person-years, accounting for 3 to 7% of dementia cases. The prevalence of LBD increases with age and ranges from 0.02 to 63.5 per 1000 persons [3]. In addition, the epidemiological characteristics of LBD differ across ancestry, which highlights the need for studies on LBD among ethnically diverse populations [4, 5]. Clinically, LBD is characterized by progressive cognitive impairment, parkinsonism, and neuropsychiatric symptoms, with extensively abnormal deposition of α-synuclein in the form of Lewy bodies, which are also featured in Parkinson’s disease (PD) pathology [6]. Unfortunately, LBD is a type of irreversible dementia with high mortality due to the lack of effective treatment [7]. Therefore, it is of great significance to probe into the complex genetic architecture of LBD, thus to better understand its underlying genetic mechanisms and investigate potential intervention targets.

Genome-wide association studies (GWAS) have successfully identified several risk loci associated with LBD [8–10]; however, the sample sizes of these GWASs are relatively small, in which the largest LBD GWAS to date only includes 2591 LBD cases and 4027 neurologically healthy individuals [10]. The smaller sample size of LBD GWAS may be presumably due to the clinical underdiagnosis or misdiagnosis of LBD. In particular, LBD typically shares features with synucleinopathies (e.g., PD) and tauopathies (e.g., AD) [11], which often brings the difficulties in clinical practice to obtain the precise diagnosis of LBD [12]. In addition, the clinically pathological diagnosis is hard to carry out, and the definitively diagnosed LBD cases generally rely on a brain autopsy after death [13, 14]. Therefore, it is infeasible to collect large samples of definitively diagnosed LBD cases in longitudinal studies. Using some state-of-the-art methods to alleviate the small sample size issue and improve the LBD GWAS power is necessary to re-explore the underlying genetic mechanisms and to provide novel insights into the biologically potential intervention targets of LBD.

Multi-trait joint analysis can borrow the correlation information from multiple correlated traits and has become a common and effective statistical tool to improve the power of the single-trait GWAS. Given the clinical and pathological overlap of LBD with AD and PD, a plausible hypothesis drawn from the neuropathological observations studies is that LBD lies on a disease continuum between AD and PD [15], and thus, one would anticipate the shared genetic underpinnings among these three diseases. More importantly, the current sample sizes of AD or PD GWASs are relatively large with substantial underlying information to exploit. Therefore, multi-trait joint analysis of LBD, AD, and PD could be more powerful not only in deeply exploring the LBD-associated genetic variants by borrowing the information from both AD and PD, but also in providing the shared pleiotropic loci among these three diseases. In addition, the shared genetic loci could also serve as intervention targets with the potential to simultaneously prevent or treat these diseases, providing critical public health and clinical significance [16]. Indeed, previous studies also supported the possible genetic overlap among these three diseases and have illustrated the potential to deeply investigate the genetic architecture of LBD through multi-trait joint analysis. For example, a recent review summarized the genetic associations for LBD, including the well-documented known loci, *APOE*, *SNCA*, and *GBA* [17]. Two additional loci *BIN1* and *TMEM175* have been recently discovered from the largest GWAS of LBD to date [10]. These loci have been implicated in AD (e.g., *APOE* and *BIN1*) as well as in PD (e.g., *SNCA*, *GBA,* and *TMEM175*), respectively, indicating the shared genetic etiology that LBD may be partly driven by the pleiotropic genetic variants associated with both AD and PD [10].

With the increase of publicly available GWAS summary data and the well-developed efficient tools, it is methodologically feasible to conduct the multi-trait joint analysis. Linkage disequilibrium score regression (LDSC) [18] is often used as an initial evaluation of the global genetic correlation among multiple traits, typically followed by some subsequent analyses. Multi-trait analysis of GWAS (MTAG) [19] resorts to the correlation of multiple related traits to improve power. However, MTAG may suffer from the inflation of false discovery rate due to the violation of the strong homogeneous assumption that all SNPs share the same variance-covariance matrix of effect sizes across traits. Association analysis based on subsets (ASSET) [20] is another flexible and powerful multi-trait method with relatively weak assumptions. ASSET could exhaustively explore all possible subsets of traits and assign an optimal one for each SNP, suggesting potential pleiotropic effects of these SNPs. Theoretically, MTAG and ASSET could complement each other.

In the present study, using the largest GWAS summary statistics of LBD, late-onset AD, and PD to date, we first performed single-trait LDSC to evaluate the quality of LBD, AD, and PD GWAS, respectively, followed by genome-wide genetic correlations with pairwise LDSC analysis. Second, we applied MTAG for LBD, AD, and PD to obtain the MTAG meta-analysis summary statistics of LBD, with additional ASSET analysis to further validate and replicate the findings from MTAG. Third, based on the results from MTAG analysis, we implemented SNP-level functional annotation to identify significant genomic risk loci followed by SNP enrichment to discover the significant functional categories of various cells or tissues involved in the development of LBD. Next, we applied conditional and joint association analysis to identify independent association signals in genomic risk loci, followed by Bayesian fine-mapping to obtain credible sets of candidate causal SNPs as well as colocalization analysis to pinpoint the shared causal variants. Finally, we performed gene-level analysis including GCTA-fastBAT and multi-tissue TWAS analysis using joint-tissue imputation (JTI) to further explore novel LBD-associated genes and reveal the underlying genetic mechanisms of LBD. Briefly, we reported the novel LBD-associated risk loci and the shared loci that may play important roles in the clinical, pathological, and genetic overlap of LBD, AD, and PD, providing novel insight into the prevention or treatment of these diseases.

## Methods

### Study design, data sources, and quality control

**Fig. 1:**
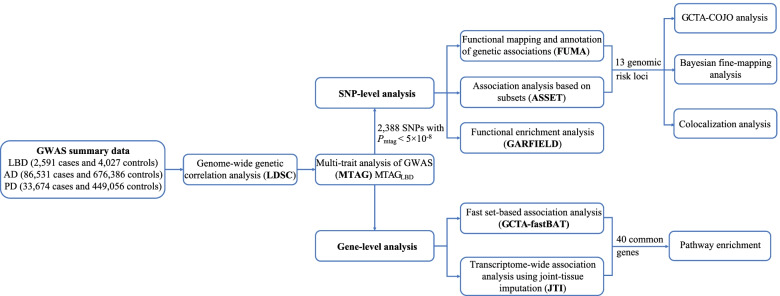
Overall study design. Pairwise genome-wide genetic correlation analysis among LBD, AD, and PD was first performed by linkage disequilibrium score regression (LDSC), followed by multi-trait meta-analysis of LBD, AD, and PD GWASs using MTAG. Based on the MTAG_LBD_ results, SNP-level analysis and gene-level analysis were further implemented to investigate the genetic basis of LBD and shared genetics underlying LBD, AD, and PD. GWAS, genome-wide association study; MTAG, multi-trait analysis of genome-wide association studies; LBD, Lewy body dementia; AD, Alzheimer’s disease; PD, Parkinson’s disease

The analysis flowchart of this study is shown in Fig. 1. We used the largest GWAS of LBD, AD, and PD to date. We obtained the largest GWAS summary statistics of LBD (2591 cases and 4027 controls) from the GWAS Catalog (https://www.ebi.ac.uk/) [10, 21], the largest late-onset AD GWAS (86,531 cases and 676,386 controls) from a large-scale meta-analysis excluding 23andMe (https://ctg.cncr.nl/software/summary_statistics) [22, 23], and the largest PD GWAS (33,674 cases and 449,056 controls) from the MRC IEU OpenGWAS database (https://gwas.mrcieu.ac.uk/) [24, 25]. These studies were all restricted to European ancestry with stringent quality control as described previously [10, 22, 24]. We converted the LBD summary statistics from human reference genome hg38 to hg19 through CrossMap (http://crossmap.sourceforge.net/) [26, 27] to ensure the same alignment reference with the other two GWAS summary statistics. In addition, we excluded the major histocompatibility complex (MHC) region (chromosome 6, 26–34 Mb) due to its complex structure, filtered SNPs with minor allele frequency (MAF) < 0.01, restricted to biallelic SNPs, and removed SNPs with duplicated or missing rs ID in each GWAS summary data for subsequent analyses. Besides, since the information including MAF, effect sizes, and standard errors of SNPs was unavailable in the AD GWAS, we estimated MAF using the 1000 Genomes Project phase 3 of European ancestry as a reference panel [28] and then estimated the effect sizes and standard errors from *Z*-scores following the previous method [29]. Detailed descriptions of all GWAS studies above were provided in Additional file 1: Table S1.

### LD score regression analysis

LD score regression (LDSC) [18, 30] is widely used for estimating the genome-wide genetic correlation between complex diseases or traits. LDSC essentially quantifies the genetic correlation by regressing the GWAS summary statistics on LD scores. We first performed the single-trait LDSC to estimate SNP-based heritability, mean *χ*^2^, genomic inflation factor *λ*_*GC*_, and the intercept for each GWAS summary statistics. Note that the mean *χ*^2^ statistic is higher in the high-LD region compared with the low-LD region and should not be further analyzed if it is less than 1.02. *λ*_*GC*_ and the intercept can be used to evaluate the polygenicity and confounding due to population stratification or cryptic relatedness.

We then conducted pairwise LDSC to estimate genome-wide genetic correlations among LBD, AD, and PD using the pre-computed LD scores of European ancestry from the 1000 Genomes Project Phase3 (https://alkesgroup.broadinstitute.org/LDSCORE/) [31]. Given that low imputation quality may lead to lower test statistics, we restricted our analysis to well-imputed HapMap3 SNPs. Bonferroni-corrected significant threshold was set at a *P* value of 0.0167 (0.05/3). Again, both single-trait and pairwise LDSC analyses can provide the overall results and direct the downstream analyses.

### Multi-trait meta-analysis with MTAG

MTAG applies generalized inverse-variance-weighted meta-analysis for multiple correlated traits and aims to detect novel genetic associations for each trait through boosting statistical power by borrowing the correlations among correlated traits [19]. Briefly, MTAG takes summary statistics from single-trait GWAS as inputs and produces trait-specific effects for one common set of SNPs. In addition, MTAG incorporates LDSC to account for sample overlap among the GWASs of multiple correlated traits [19]. MTAG relies on the key homogeneous assumption that all SNPs across traits share the same variance-covariance matrix of effect sizes, but the estimator of MTAG can be still consistent even if this assumption is violated when some SNPs influence only a subset of the traits [19]. Another important feature of MTAG is that the summary statistics obtained from MTAG for each trait can be used like summary statistics from a single-trait GWAS.

We denote the summary statistics from single-trait GWAS as GWAS_LBD_, GWAS_AD_, and GWAS_PD_, respectively, and the summary statistics of LBD from MTAG analysis as MTAG_LBD_. The genome-wide significance level for MTAG_LBD_ was set at *P*_mtag_ < 5 × 10^−8^. We performed an analysis using MTAG v.1.0.8 and calculated the maximum false discovery rate (maxFDR) to evaluate the overall inflation due to violation of the homogeneous assumption [19].

### Cross-trait meta-analysis with ASSET

ASSET builds on a generalized fixed-effects meta-analysis framework, allows SNPs affecting only a subset of analyzed traits, and is robust to heterogeneous genetic effects and sample overlap among multiple correlated traits [20]. ASSET exhaustively explores all possible subsets of traits for each given SNP and determines the optimal trait subset [20]. Given that MTAG is unable to account for pleiotropic effects of individual SNPs at the phenotypic level, we further carried out ASSET analysis to verify the significant genetic associations of MTAG_LBD_ and to identify the optimal trait subset for each significant SNP. The SNPs used in ASSET analysis were extracted from three single-trait GWAS summary statistics, with effect directions all aligned to the effect alleles of GWAS_LBD_. Here, we not only adopted a one-sided ASSET procedure assuming the same direction of association for all three diseases but also a two-sided ASSET analysis allowing the associations with opposite directions, to fully investigate the different association directions of SNPs on these diseases. SNPs with both *P*_mtag_ and *P*_asset_ less than 5 × 10^−8^ were considered to be further verified if these SNPs are also in the optimal trait subset including LBD.

### SNP annotation using FUMA

FUMA v1.3.6b [32], an online platform at https://fuma.ctglab.nl/ [33], was applied to annotate genome-wide significant SNPs of MTAG_LBD_. We performed FUMA annotation with default settings and used the 1000 Genomes Project Phase3 of European ancestry as a reference panel. SNPs with *P* < 5 × 10^−8^ and independent from each other at *r*^2^ < 0.6 within 1 Mb were defined as independent significant SNPs. Lead SNPs, a subset of the independent significant SNPs, were defined if they are independent from each other at *r*^2^ < 0.1. Genomic risk loci were identified by merging the LD blocks of independent significant SNPs that are closely located to each other (< 250 kb) [32]. The top lead SNP was defined as the SNP with the lowest *P* value in a specific region. Functional annotations, including ANNOVAR categories [34], combined annotation-dependent depletion (CADD) [35] scores, and RegulomeDB scores [36], were also obtained by FUMA [32]. In addition, genome-wide significant SNPs from GWAS_LBD_ were also annotated by FUMA for comparison.

### Conditional analysis using GCTA-COJO

To further investigate whether the genomic risk loci of MTAG_LBD_ contain multiple independent signals, we performed conditional and joint association analysis using the stepwise model selection procedure in GCTA-COJO [37, 38]. Only independent SNPs from FUMA (*P*_mtag_ < 5×10^−8^) in the genomic risk loci were included in GCTA-COJO analysis, with additional signals being reported when joint *P* value < 5 × 10^−8^. Again, 1000 Genomes Project Phase3 of European ancestry was used as the reference panel for estimating LD.

### Bayesian fine-mapping analysis

We applied Bayesian fine-mapping analysis to obtain the SNP credible sets for each locus analyzed in GCTA-COJO analysis using the finemap.abf function in coloc v5 R package (https://chr1swallace.github.io/coloc/) under default settings [39]. With posterior probability (PP) of each SNP being causal provided in each locus, the 90% credible set of SNPs in a certain locus was obtained by inclusion of SNPs according to the PP order until the cumulative posterior probability up to 0.90. The SNP credible set would produce the potential candidate causal SNPs.

### Colocalization analysis

The Bayesian colocalization method requires a single causal variant assumption for each trait in a specific analyzed region [40]. The hypothesis H3 that both traits are associated but with distinct causal variants and H4 that both traits are associated and share a single causal variant are of interest in our analysis. The posterior probability of H3 and H4 is denoted as PP3 and PP4, respectively, and the threshold for causal signals was set at PP3 or PP4 > 0.75. Using the coloc.abf function in the coloc v5 R package [39] with prior probabilities all set at 1 × 10^−4^, colocalization analysis was performed on all SNPs in each locus identified by FUMA to further colocalize causal variants between LBD and AD as well as LBD and PD.

### Functional enrichment analysis

We performed a detailed functional enrichment analysis using GARFIELD [41]. GARFIELD leverages GWAS summary statistics and various regulatory/functional annotations, including genic annotations, histone modifications, transcription factor binding sites, chromatin segmentation states, and open chromatin data (FAIRE, DHS Hotspots, peaks, and footprints) in various cells or tissues to find out the characteristics relevant to a trait of interest under different GWAS *P* value thresholds [41]. Briefly, given GWAS summary statistics and functional annotations (https://www.ebi.ac.uk/birney-srv/GARFIELD/) [42], GARFIELD first performs the LD pruning with a greedy procedure to extract independent SNPs from genome-wide genetic associations, followed by LD tagging annotation to annotate each variant with regulatory annotations. Then, utilizing a logistic regression model, GARFIELD calculates enrichment statistics (odds ratios, OR) and *P* values at user-defined GWAS *P* value thresholds for each annotation. We assessed the enrichment of significant SNPs in MTAG_LBD_ at the Bonferroni-corrected significance level *P* < 4.98 × 10^−5^ (0.05/1005), with 1005 being the number of annotations.

### Gene-level analysis

Using different methods with different model assumptions to obtain the overlapped signals can avoid the risk of false discoveries from using a single method. Therefore, we applied two gene-level approaches with distinct principles, GCTA-fastBAT and TWAS, as parallel analyses to obtain the common LBD-associated genes for subsequent pathway analysis.

GCTA-fastBAT is a fast set-based association analysis widely applied in the gene-based analysis [38, 43]. GCTA-fastBAT integrates *z*-statistics from a set of SNPs within a specific genomic region into a quadratic form of a multivariate normal variable and then calculates *P* values from an approximated distribution of the sum of *χ*^2^ statistics over the SNPs, while accounting for LD between SNPs. To identify candidate genes, we here conducted a gene-based analysis using MTAG_LBD_ for all 24,763 genes by GCTA-fastBAT. Only SNPs located within the gene were included to examine the gene-trait associations. LD information from 1000 Genomes Project Phase3 was utilized in the gene-based analysis. The genome-wide Bonferroni-corrected significance level was set as *P*_fastBAT_ < 2.02 × 10^−6^ (0.05/24,763).

Transcriptome-wide association studies (TWAS) aim to integrate GWAS and eQTL studies to identify tissue-specific gene-trait associations [44, 45]. We used MTAG_LBD_ results and the S-PrediXcan program [46] combined with joint tissue imputation (JTI) models to perform a two-stage TWAS analysis. As an extension of PrediXcan [44], the JTI method substantially improves prediction performance by leveraging shared expression regulation and epigenetic similarity among different tissues [47]. We used JTI models in 13 different regions of brain tissue derived from the Genotype-Tissue Expression project version 8 (GTEx v8) transcriptome data [48], with Bonferroni correction for multiple testing in each tissue.

### Pathway enrichment

To understand the biological mechanisms of the significant candidate genes identified from MTAG_LBD_, we performed pathway enrichment using g:Profiler [49]. Significant pathways were declared with a Bonferroni-corrected significance level (adjusted *P* < 0.05).

### Data visualization

R package CMplot (https://github.com/YinLiLin/CMplot) was used for producing Manhattan plots [50, 51]. LocusZoom (http://locuszoom.org/) was used for locus visualization [52, 53]. Other visualizations were performed in R.

## Results

### Linkage disequilibrium score regression

Single-trait LDSC estimates for GWAS_LBD_, GWAS_AD_, and GWAS_PD_ were shown in Additional file 1: Table S2. The estimates of liability-scale SNP heritability were 0.1122 (se = 0.0528) for LBD, 0.0105 (se = 0.0017) for AD, and 0.0259 (se = 0.0024) for PD. The mean χ^2^ statistics were all greater than 1.02, the genomic inflation factors (*λ*_*GC*_) were all less than 1.1, and the LDSC intercepts were all close to 1. All these results indicated that the inflation of test statistics was probably caused by polygenicity rather than potential population stratification.

Pairwise LDSC analysis found positive genome-wide genetic correlations between LBD and AD (rg = 0.6603, se = 0.2001; *P* = 0.0010), between LBD and PD (rg = 0.6352, se = 0.1880; *P* = 0.0007), and between AD and PD (rg = 0.2136, se = 0.0860; *P* = 0.0130) (Table 1).

**Table 1:** Pairwise genetic correlation among LBD, AD, and PD using LDSC

Trait1	Trait2	rg	se	***P***
LBD	PD	0.6352	0.1880	0.0007*
LBD	AD	0.6603	0.2001	0.0010*
AD	PD	0.2136	0.0860	0.0130*

*LBD* Lewy body dementia, *AD* Alzheimer’s disease, *PD* Parkinson’s disease, *rg* genetic correlation estimate, *se* standard error

*P value reached the Bonferroni-corrected significance level *P* < 0.0167 (0.05/3)

### MTAG analysis and LBD-associated loci discovery

**Fig. 2:**
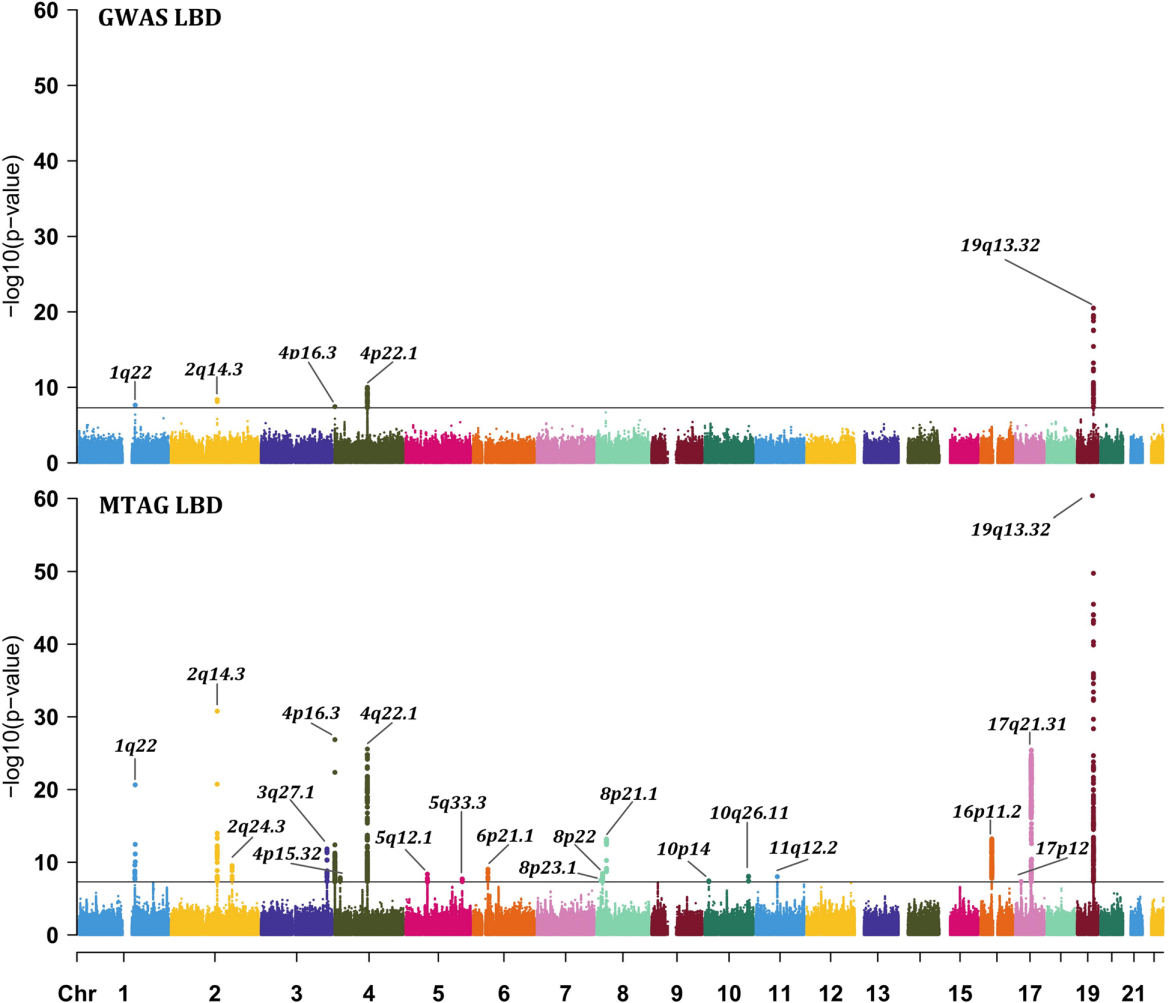
Manhattan plots of GWAS_LBD_ (**a**) and MTAG_LBD_ (**b**). The *x*-axis denotes the chromosomal position, and the *y*-axis shows the −log10 *P* value. The horizontal black line corresponds to the genome-wide significance threshold (*P* < 5 × 10^−8^). Labels are the chromosome regions where genomic risk loci are located. Note that the Manhattan plots were plotted at *P* values truncated by 1 × 10^−60^ for better visualization

We performed a meta-analysis of GWAS_LBD_, GWAS_AD_, and GWAS_PD_ using MTAG. A total of 5,103,377 SNPs were available for MTAG meta-analysis, among which 2388 SNPs in MTAG_LBD_ reached the genome-wide significance level (*P*_mtag_ < 5 × 10^−8^, excluding the MHC region). All 2388 genome-wide significant SNPs along with their corresponding FUMA annotations were provided in Additional file 1: Table S3. The Manhattan plots were shown in Fig. 2. From GWAS_LBD_ to MTAG_LBD_, the mean *χ*^2^ statistic increased from 1.024 to 1.132, the genomic risk loci increased from 5 to 20 (Additional file 1: Table S4 and Table 2), and the total number of lead SNPs (*P*_mtag_ < 5 × 10^−8^ and *r*^2^ < 0.1) increased from 10 to 43 (Additional file 1: Table S5). All the results were expected since MTAG analysis, by borrowing the correlation among multiple traits, should be more powerful than single-trait analysis. The genomic inflation factor *λ*_GC_ of MTAG_LBD_ was 1.061. The maxFDR for MTAG_LBD_ was 0.024, suggesting no overall inflation due to violation of the homogeneous assumption.

**Table 2:** Genomic risk loci and corresponding top variants identified by FUMA using SNPs with *P*_mtag_ < 5 × 10^−8^ in MTAG_LBD_

Top SNP	Genomic risk loci	Chromosome loci	n.sig	***P***_**gwas**_	***P***_**mtag**_	***P***_**asset**_	Trait subset	Variant annotion	nearestGene
rs35603727	1:155024309–156300731	1q22	11	4.06 × 10^−7^	2.25 × 10^−21^	1.67 × 10^−20^	LBD, PD	Intronic	*UBQLN4*
rs6733839	2:127839474–127894851	2q14.3	41	4.16 × 10^−9^	1.64 × 10^−31^	5.20 × 10^−37^	LBD, AD	Intergenic	*BIN1*
rs1474055	2:169091942–169166282	2q24.3	7	6.32 × 10^−1^	2.97 × 10^−10^	7.96 × 10^−12^	PD	Intergenic	*STK39*
rs10513789	3:182704808–182833363	3q27.1	27	9.88 × 10^−1^	1.44 × 10^−12^	2.15 × 10^−12^	PD	Intronic	*MCCC1*
rs34311866	4:819789–1030779	4p16.3	83	1.40 × 10^−6^	1.35 × 10^−27^	1.18 × 10^−25^	LBD, PD	Exonic	*TMEM175*
rs4389574	4:15706502–15743332	4p15.32	6	9.02 × 10^−1^	1.37 × 10^−8^	1.04 × 10^−12^	PD	Intronic	*BST1*
rs1372518	4:90513519–91127134	4q22.1	215	1.01 × 10^−7^	2.66 × 10^−26^	1.17 × 10^−32^	LBD, PD	UTR5	*SNCA*
rs1867598	5:60011636–60489094	5q12.1	16	1.21 × 10^−1^	4.34 × 10^−9^	1.87 × 10^−12^	LBD, PD	Intronic	*ELOVL7*
rs9790947	5:156506352–156568510	5q33.3	4	3.60 × 10^−1^	1.98 × 10^−8^	3.37 × 10^−9^	LBD, AD, PD	Intronic	*HAVCR2*
rs13216201	6:41140984–41166310	6p21.1	6	7.14 × 10^−2^	9.18 × 10^−10^	1.41 × 10^−10^	LBD, AD, PD	Intronic	*TREML2*
rs4731	8:11666337–11674897	8p23.1	1	2.04 × 10^−2^	3.09 × 10^−8^	6.25 × 10^−8^	LBD, AD, PD	Exonic	*FDFT1*
rs620490	8:16695418–16739127	8p22	15	5.56 × 10^−1^	3.41 × 10^−9^	4.29 × 10^−9^	PD	ncRNA_intronic	*RP11-13N12.1*
rs1532278	8:27456253–27468862	8p21.1	12	9.05 × 10^−3^	6.35 × 10^−14^	2.24 × 10^−21^	LBD, AD, PD	Intronic	*CLU*
rs11257240	10:11707563–11720620	10p14	3	9.92 × 10^−2^	3.60 × 10^−8^	6.48 × 10^−11^	LBD, AD, PD	Intergenic	*RP11-138I18.2*
rs117896735	10:121372328–121710488	10q26.1	4	9.63 × 10^−1^	8.46 × 10^−9^	1.94 × 10^−9^	PD	Intronic	*INPP5F*
rs12453	11:59856028–60041296	11q12.2	1	2.16 × 10^−1^	9.52 × 10^−9^	2.72 × 10^−27^	LBD, AD	Exonic	*MS4A6A*
rs7185007	16:30571910–31155458	16p11.2	145	6.65 × 10^−3^	6.10 × 10^−14^	1.05 × 10^−11^	LBD, AD, PD	Intergenic	*FBXL19-AS1*
rs6502490	17:15868291–16240507	17p12	1	4.13 × 10^−2^	4.46 × 10^−8^	6.19 × 10^−8^	LBD, PD	Intronic	*NCOR1*
rs2532307	17:43460181–44865603	17q21.31	1605	4.83 × 10^−2^	3.84 × 10^−26^	3.68 × 10^−21^	LBD, PD	Intronic	*KANSL1*
rs157595	19:45192348–45734751	19q13.32	185	1.58 × 10^−19^	4.24 × 10^−145^	1.39 × 10^−205^	LBD, AD	Intergenic	*APOC1*

Genomic risk loci are identified by FUMA merging the LD blocks of independent significant SNPs that are closely located to each other (< 250 kb); chromosome loci represent the chromosomal regions containing genomic risk loci; n.sig represents the number of SNPs with *P*_mtag_ < 5 × 10^−8^ in the locus; trait subset is the optimal trait subset of top SNP identified by ASSET

### SNP-level analysis

#### ASSET analysis and SNP-based pleiotropy

In one-sided ASSET analysis, 2160 of 2388 significant SNPs in MTAG analysis (90.45%) were verified in ASSET analysis (Additional file 1: Table S3), two-sided ASSET analysis illustrated that no SNPs were both positively and negatively associated with the subset of traits (Additional file 1: Table S3). Among these 2160 verified SNPs, 1880 SNPs (about 87.04%) were included in the trait subset {LBD, PD}, followed by 185 SNPs (about 8.56%) in the trait subset {LBD, AD}, and 95 (4.40%) SNPs in the trait subset {LBD, AD, PD}. Note that most SNPs identified for the trait subset {LBD, PD} were in the 17q21.31 region, whose complex LD structure can result in a relatively long genomic risk locus (Table 2), thus leading to much more SNPs being included in the set of LBD and PD than the set of LBD and AD. Notably, no SNPs were included in the trait subset {LBD}, indicating that most of the observed significant SNPs in MTAG_LBD_ may probably be the potential pleiotropic SNPs shared with AD and/or PD.

We further summarized the number and proportion of 2160 verified SNPs in each subset of traits for 20 genomic risk loci (Table 3). Those loci in which both the majority of confirmed SNPs as well as the top SNPs can be assigned to a specific subset of traits, were considered to be potential pleiotropic loci. For loci 2q14.3, 11q12.2, and 19q13.32, the majority of confirmed SNPs were in the trait subset {LBD, AD} and the top SNPs in these loci were also confirmed with an optimal trait subset {LBD, AD}, suggesting these three loci may be the pleiotropic loci between LBD and AD. Especially in 19q13.32, the optimal trait subset for all confirmed SNPs was {LBD, AD}. For loci 1q22, 4p16.3, 4q22.1, 5q12.1, and 17q21.31, the majority of confirmed SNPs were in the trait subset {LBD, PD}, and the top SNPs in these loci were also confirmed with the optimal trait subset {LBD, PD}, suggesting these five loci may be the pleiotropic loci between LBD and PD. Especially in loci 4q22.1 and 5q12.1, the optimal trait subset for all confirmed SNPs was {LBD, PD}. The dominant trait subset as well as the optimal trait subset for top SNPs at 5q33.3, 6p21.1, 8p21.1, 10p14, and 16p11.2 was {LBD, AD, PD}, which may provide insights into the overlapping etiology and pathogenesis for all three diseases. Note that none of the confirmed SNPs is located in loci 2q24.3, 4p15.32, 10q26.1, 11q25, and 12q24.31. All confirmed SNPs at 3q27.1 and 8p22 were included in trait subset {LBD, PD}; nevertheless, the corresponding top SNPs, rs10513789 and rs620490, were included in optimal trait subset {PD}.

**Table 3:** The number of verified SNPs in each subset of traits

Chromosome loci	SNP number	SNP number in each subset of traits			
		LBD	LBD, AD	LBD, PD	LBD, AD, PD
1q22	7	–	–	6	1
2q14.3	41	–	29	–	12
4p16.3	74	–	–	71	3
4q22.1	189	–	–	189	–
5q12.1	16	–	–	16	–
5q33.3	4	–	–	–	4
6p21.1	6	–	–	–	6
8p21.1	12	–	3	–	9
10p14	3	–	–	–	3
11q12.2	1	–	1	–	–
16p11.2	81	–	–	36	45
17q21.31	1567	–	–	1555	12
19q13.32	152	–	152	–	–

The unverified loci including 2q24.3, 3q27.1, 4p15.32, 8p22, 8p23.1, 10q26.1, and 17p12 were not listed

“–” represents no SNP was significant (*P*_asset_ < 5 × 10^−8^) in this subset of traits

*LBD* Lewy body dementia, *AD* Alzheimer’s disease, *PD* Parkinson’s disease

In summary, we confirmed 13 genomic risk loci significantly associated with LBD, 3 were likely to be shared with AD, 5 shared with PD, and 5 shared with both AD and PD. The heritability explained by 13 top SNPs of these loci was estimated to be 0.70%, which could account for 6.24% (0.0070/0.1122) of the overall heritability of LBD. Specifically, 5 out of these 13 loci overlapped with that identified from GWAS_LBD_, and genes closest to top SNPs in these loci were *APOC1*, *BIN1*, *SNCA*, *TMEM175*, and *UBQLN4*. Eight loci have not been found to be associated with LBD in previous GWAS_LBD_, and top SNPs in these regions were mapped to genes *CLU*, *ELOVL7*, *FDFT1*, *FBXL19-AS1*, *HAVCR2*, *KANSL1*, *NCOR1*, and *TREML2*.

#### Functional annotations

We summarized the variant annotations through FUMA for 2160 SNPs with both *P*_mtag_ and *P*_asset_ less than 5 × 10^−8^ (Additional file 1: Table S6). Most variants (92.36%) were located in non-coding regions, such as intronic and intergenic regions; only a few SNPs were exonic variants, including 46 (2.13%) exonic variants of coding RNA and 31 (1.44%) exonic variants of non-coding RNA. The most significant exonic variant of coding RNA was rs112849259 (*P*_mtag_ = 1.16 × 10^−83^, mapped gene: *TOMM40*) in 19q13.32 locus, followed by rs7412 (*P*_mtag_ = 6.99 × 10^−44^, mapped gene: *APOE*) in 19q13.32 locus, and rs34311866 (*P*_mtag_ = 1.35 × 10^−27^, mapped gene: *TMEM175*) in 4p16.3 locus. The exonic variant of non-coding RNA in 4q22.1 locus, rs2245801, was significant in both GWAS_LBD_ and MTAG_LBD_ (*P*_gwas_ = 3.06 × 10^−8^, *P*_mtag_ = 4.17 × 10^−22^, mapped gene: *SNCA*-*AS1*). The variant with the highest CADD score was rs17651549 (*P*_mtag_ = 1.69 × 10^−24^, CADD score 26.8) in 17q21.31 locus, which is an exonic variant of *MAPT*. RegulomeDB scores showed that the variant rs17572495 (*P*_mtag_ = 1.36 × 10^−23^) had a relatively higher regulation level, which was also in the 17q21.31 locus located in the 5′UTR of *MAPT*. Note that both rs17651549 and rs17572495 were verified to be in the optimal subset of trait {LBD, PD} from the ASSET analysis, which further highlighted the role of *MAPT* in the shared genetic etiology of LBD and PD.

#### Independent signals within loci

We performed GCTA-COJO analysis in 13 verified MTAG_LBD_-associated loci. In addition to the 4q22.1 and 19q13.32 loci, no additional independent SNPs were identified in other loci after conditioning on the top variant (Additional file 1: Table S7). Of interest, 1555 SNPs in 17q21.31 were included in optimal trait subset {LBD, PD} in ASSET analysis, but only the top SNP rs2532307 was identified as an independent signal. Besides, the additional two independent SNPs in the 4q22.1 region were rs11931074 (conditional *P* = 1.74 × 10^−20^) and rs356177 (conditional *P* = 2.64 × 10^−10^). Ten additional independent signals were identified in the *APOE* locus, which was presumably due to its complex LD structure, highlighting the significance of this region to the co-pathology of LBD and AD.

#### SNP credible sets within loci

A total of 1111 SNPs in 90% credible sets were identified for 13 genomic risk loci (Additional file 1: Table S8). Among the 5 loci identified in GWAS_LBD_, 90% credible sets of 4 loci (1q22, 2q14.3, 4p16.3, and 19q13.32) contained only the top SNP (PP > 0.99). While in the 4q22.1 locus, five SNPs were identified in its 90% credible set with the top SNP rs1372518 (PP = 0.56, mapped gene: *SNCA*) included. For another 8 loci, there were multiple SNPs in their 90% credible sets. For example, 17q21.31 locus, in which only one independent signal was identified by GCTA-COJO, there were as many as 966 SNPs in the 90% credible set.

#### Colocalization analysis

Among the 13 genomic risk loci, colocalization analysis totally identified 7 loci with PP3 or PP4 larger than 0.75 (Table 4). Three loci (2q14.3, 8p21.1, and 19q13.32) were suggested to share the same causal variant between LBD and AD. For 8p21.1 locus, the optimal trait subset was {LBD, AD, PD} from the ASSET analysis; however, colocalization analysis found shared causal variant between LBD and AD (PP4 = 0.8264) rather than LBD and PD (PP4 = 0.0400), with top SNP identified as potential shared causal variant (rs1532278, PP4 = 0.2850, mapped gene: *CLU*). Besides, both 1q22 (PP4 = 0.9893) and 4p16.3 (PP4 = 0.9976) were suggested to share the same causal variant between LBD and PD. The 4q22.1 locus with PP3 larger than 0.75 was suggested to share distinct causal SNPs between LBD and PD. Besides, the 16p11.2 locus was colocalized between LBD and AD as well as LBD and PD, in line with the findings from the ASSET analysis that the dominant trait subset of this locus was {LBD, AD, PD}. In addition, for these 13 genomic risk loci, the PP4 of each SNP in each locus were provided in Additional file 1: Table S9; the SNP with the maximum PP4 was considered as the most likely shared causal variant. The LocusZoom plots were displayed in Additional file 2: Figs. S1-S13.

**Table 4:** Summary of colocalization results in 13 genomic risk loci

Chromosome loci	LBD-AD			LBD-PD		
	PP3	PP4	Best Causal	PP3	PP4	Best causal
1q22	0.0064	0.0370	rs35603727^b^	0.0105	0.9893^†^	rs35603727^b^
2q14.3	0.0002	0.9998^a^	rs4663105	0.0198	0.1529	rs4663105
4p16.3	0.0213	0.4839	rs34311866^b^	0.0023	0.9976^a^	rs34311866^b^
4q22.1	0.0432	0.5154	rs7680557	1.0000^a^	0.0000	rs356203
5q12.1	0.0001	0.0012	rs4647170	0.0212	0.3983	rs75646569
5q33.3	0.0023	0.1630	rs6555853	0.0002	0.0168	rs9790947^b^
6p21.1	0.0014	0.2463	rs34346157	0.0002	0.0602	rs13216201^b^
8p21.1	0.0009	0.8264^a^	rs1532278^b^	0.0000	0.0400	rs1532276
10p14	0.0012	0.4398	rs7920721	0.0000	0.0021	rs11257240^b^
11q12.2	0.0070	0.2948	rs7926354	0.0001	0.0033	rs11512743
16p11.2	0.0809	0.7554^a^	rs889555	0.1608	0.7843^a^	rs8050588
17q21.31	0.1041	0.4977	rs2532276	0.1068	0.5140	rs58879558
19q13.32	0.0208	0.9792^a^	rs114536010	0.0277	0.0984	rs157595^b^

*LBD* Lewy body dementia, *AD* Alzheimer’s disease, *PD* Parkinson’s disease, *PP* posterior probability; *PP3* PP of both traits are associated but with distinct causal variants, *PP4* PP of both traits are associated and share a single causal variant, *Best causal* SNP with the highest PP4 is considered to be the causal variant in the genomic risk loci

^a^PP3 or PP4 larger than 0.75

^b^The potential causal SNP was the top SNP in the locus

#### Functional enrichment analysis

**Fig. 3:**
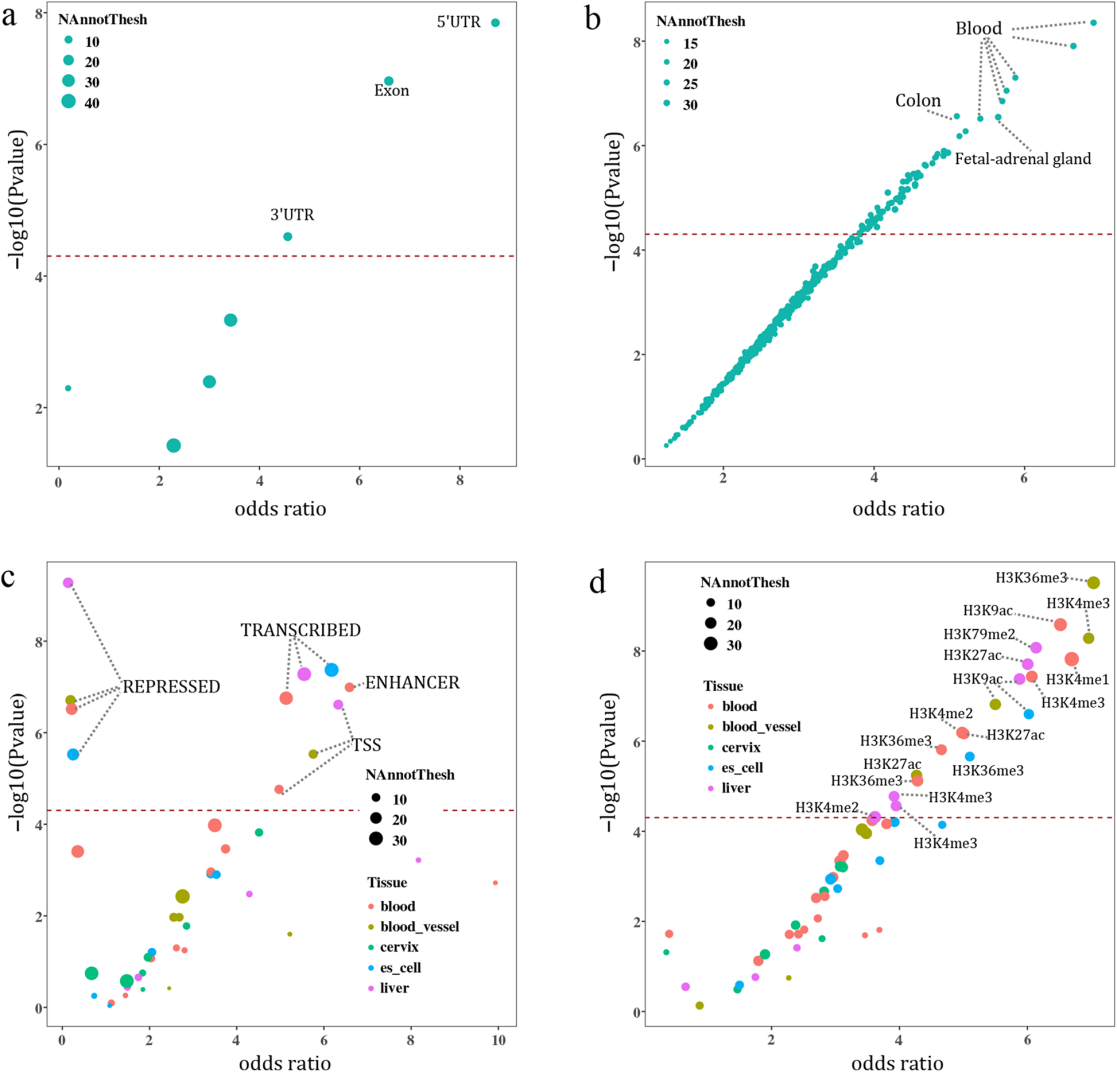
GARFIELD enrichment of SNPs with *P*_mtag_ < 5 × 10^−8^ in MTAG_LBD_. Enrichment in genic regions (**a**), DHS (hotspots) regions of different tissues (**b**), chromatin states of different tissues (**c**), and histone modified regions of different tissues (**d**). The horizontal axis represents the enrichment odds ratios of each annotation category derived from logistic regression, and the vertical axis shows the corresponding −log10 *P* values. The dashed line corresponds to the significance threshold of *P* = 0.05/1005. The size of the dots indicates the number of independent SNPs in a specific annotation. The color of the dots in **c** and **d** indicates different tissue types

Enrichment analysis results of GARFIELD were shown in Additional file 1: Table S10. We observed significant enrichment of significant SNPs from MTAG_LBD_ in several regulatory and functional categories: (1) SNP enrichment in genic regions suggested that these SNPs were significantly enriched in exon region (OR = 6.58, *P* = 1.09 × 10^−7^) (Fig. 3a); (2) SNPs located at DNase I hypersensitive sites showed highly significant enrichment in several tissues, with colon identified as the most significant enrichment (OR = 5.10, *P* = 2.74 × 10^−7^) (Fig. 3b); (3) SNP enrichment in different chromatin state associated regions revealed that the transcribed regions were significantly enriched in different tissues (embryonic stem cell: OR = 6.18, *P* = 4.25 × 10^−8^; liver: OR = 5.55, *P* = 5.26 × 10^−8^; blood: OR = 5.13, *P* = 1.76 × 10^−7^) (Fig. 3c). Significant enrichment was also found in enhancers of blood (OR = 6.59, *P* = 1.02 × 10^−7^). Interestingly, although the repressed regions were also significantly enriched, their odds ratios were all less than one; and (4) SNP enrichment in regulatory regions determined by histone modifications showed significant enrichment in distinct cells or tissues (Fig. 3d). The most significantly enriched histone marker was H3K36me3 in the blood vessel (OR = 7.04, *P* = 3.06 × 10^−10^), which has been confirmed to be associated with transcribed portions of genes.

### Gene-level analysis

#### Prioritization of candidate genes

Overall, 69 candidate genes were identified to be associated with LBD at the significance level *P*_fastBAT_ < 2.02 × 10^−6^ (Additional file 1: Table S11). A total of 110,760 tissue-specific genes were included in TWAS analysis, and 467 tissue-specific genes (98 unique genes) were identified at tissue-specific Bonferroni-corrected significance level (Additional file 1: Table S12). Finally, 40 genes were commonly identified by both GCTA-fastBAT and TWAS analyses (Additional file 1: Table S13), including *APOC1* (*P*_fastBAT_ = 1.02 × 10^−120^, *P*_TWAS_ = 2.38 × 10^−8^), *SNCA* (*P*_fastBAT_ = 5.20 × 10^−35^, *P*_TWAS_ = 1.82 × 10^−11^), *TMEM175* (*P*_fastBAT_ = 1.38 × 10^−24^, *P*_TWAS_ = 1.21 × 10^−11^), *MAPT* (*P*_fastBAT_ = 3.76 × 10^−19^, *P*_TWAS_ = 3.72 × 10^−18^), *CLU* (*P*_fastBAT_ = 6.25 × 10^−10^, *P*_TWAS_ = 1.23 × 10^−8^), and *FBXL19* (*P*_fastBAT_ = 4.73 × 10^−15^, *P*_TWAS_ = 5.47 × 10^−14^).

#### Pathway enrichment

We used g:Profiler to perform pathway enrichment analysis for 40 candidate genes commonly detected by both GCTA-fastBAT and TWAS analyses. A total of 31 significantly enriched pathways were identified (Additional file 1: Table S14) at a Bonferroni-corrected significance level (adjusted *P* < 0.05). These pathways were primarily synaptic-vesicle function, protein and lipid-related, such as vesicle-mediated transport (GO:0016192, adjusted *P* = 2.89 × 10^−3^), synaptic vesicle cycle (GO:0099504, adjusted *P* = 1.25 × 10^−2^), neurofibrillary tangle assembly (GO:1902988, adjusted *P* = 1.55 × 10^−2^), and protein-lipid complex (GO:0032994, adjusted *P* = 1.33 × 10^−2^).

## Discussion

In the present study, using the largest LBD, AD, and PD GWAS summary data to date, we performed a comprehensive large-scale genome-wide cross-trait analysis, followed by various SNP-level and gene-level genetic approaches, to deeply investigate the genetic architecture of LBD as well as the shared genetic etiology of LBD, AD, and PD. Overall, we found a significant positive genome-wide genetic correlation between LBD and AD, LBD and PD, and AD and PD. The genetic correlation was highest between LBD and AD, followed by between LBD and PD and between AD and PD. Various multi-trait analyses identified 13 common genetic loci for LBD including 5 previously reported loci (1q22, 2q14.3, 4p16.3, 4q22.1, 19q13.32) and 8 novel biologically plausible genetic associations (5q12.1, 5q33.3, 6p21.1, 8p23.1, 8p21.1, 16p11.2, 17p12, 17q21.31), among which *APOC1* (19q13.32), *SNCA* (4q22.1), *TMEM175* (4p16.3), *CLU* (8p21.1), *MAPT* (17q21.31), and *FBXL19* (16p11.2) were also identified by gene-level analysis. In addition to focusing on *cis*-regulation of genetic variants on proximal genes, we have also searched the large-scale whole blood trans-eQTL summary statistics from eQTLGen [54] to explore the trans-regulation evidence of the top SNP in each locus, while no SNPs were found to be trans-eQTL.

Of note, the regulatory mechanisms underlying LBD seem to be distinct and more complex in the locus 4q22.1 compared with that underlying AD and PD. Taking the locus 4q22.1 as an example, for MTAG_LBD_, the coding gene *SNCA* was mapped by the top SNP rs1372518 located at the 5′UTR of *SNCA*, but for GWAS_LBD_, the top SNP in this locus, rs7680557, was close to the gene *SNCA-AS1*, which overlaps with 5′UTR of *SNCA* and is well-known to regulate *SNCA* expression by encoding a long non-coding antisense RNA [9]. In addition, another independent SNP rs11931074, which is an intron variant of the gene *SNCA* with its polymorphism being suggested to be associated with PD [55, 56], was identified in locus 4q22.1 from GCTA-COJO analysis. Colocalization analysis also highlighted this locus with distinct causal SNPs between LBD and PD, suggesting the potentially different roles of this locus in the pathogenesis of PD and LBD [10].

Functional enrichment analysis illustrated that LBD-associated variants were mainly enriched in regions relevant to gene transcription and activation, such as exon regions, transcribed region enhancers, and histone marker H3K36me3. Interestingly, tissue enrichment analysis based on DHS annotation showed that gastrointestinal tissues, including the colon and small intestine, had a high degree of enrichment. A pathoanatomical study of LBD has found that alpha-synuclein aggregated in the distal esophagus, stomach, and colon [57]. Braak et al. hypothesized that abnormal alpha-synuclein accumulation would begin in the gut and further progress to the brain in a prion-like manner through the vagus nerve, which has been confirmed by animal experiments [58–60].

Both the SNP-level analysis and gene-level analysis converged on the same relevant risk loci, the same potential causal variants as well as the same risk genes, including previously discovered genes associated with LBD (*SNCA* [4q22.1], *APOC1* [19q13.32]), and three potential novel genes *CLU* (8p21.1), *MAPT* (17q21.31), and *FBXL19* (16p11.2)*.CLU*, which encodes clusterin, a glycoprotein associated with AD, binds α-synuclein aggregated species and is present in Lewy bodies, intraneuronal aggregates mainly composed of fibrillary α-synuclein [61, 62]. A recent experimental study suggested that extracellular clusterin blocks the binding site of α-synuclein fibrils, limits the uptake of α-synuclein fibrils by astrocytes, then probably leads to aggregation of clusterin and formation of Lewy bodies, and hence, contributes to the α-synucleinopathy [61]. By querying the super-enhancer database (SEdb) [63, 64], we found that the top SNP rs1532278 in locus 8p21.1 was located in super-enhancers of multiple tissues, including the dorsolateral-prefrontal cortex, H1-hESC cell, and intestine. These super-enhancers overlap with the *CLU* region and are closely related to the activation of *CLU*, suggesting the role of enhancers in the pathogenesis of LBD.

*MAPT*, the gene encoding microtubule-associated protein tau, is well-established known to play a critical role in tauopathies implicated in AD [65, 66]. *MAPT* is characterized by two main haplotypes, termed H1 and H2; a previous study has indicated the role of H1G in susceptibility to dementia with Lewy Bodies [67]. In addition, an animal study suggested that reducing tau does not affect α-synuclein expression and does not prevent α-synuclein inclusion formation [68]. Another animal study suggested that targeting tau oligomers benefits a mouse model of α-synucleinopathy with protection from cognitive and motor deficits, decrease of toxic tau oligomers levels [69]. These animal studies suggested that tau may be occurring downstream or independent of the pathological conversion of α-synuclein and may be a viable therapy for treating diseases with the interaction of tau and α-synuclein like LBD [69].

*FBXL19* encodes a member of the Skp1-Cullin-F-box family of E3 ubiquitin ligases which could regulate the ubiquitination and degradation of inflammatory cytokines, such as interleukin (IL)-1β, IL-33, and tumor necrosis factor-α (TNF-α). Previous studies have suggested that the upregulation of pro-inflammatory cytokines plays different roles in both neurodegeneration and neuroprotection [70, 71]. Besides, understanding the pro-inflammatory cytokine signaling pathways involved in the regulation of AD is significant for the development of therapeutic strategies [71]. For example, IL-33 signaling has been demonstrated to play diverse but significant roles in the homeostasis of the central nervous system diseases such as neurodegenerative diseases [71]. *FBXL19* protein could serve as a negative regulator to inhibit the IL-33-mediated signaling by regulating the ubiquitination and degradation with a potential neuroprotection effect [71, 72].

Pathway enrichment analysis suggested an important role of pathways involving synaptic vesicle function, neurofibrillary tangle, and lipids. The α-synuclein pathology was confirmed to be featured by both LBD and PD [73, 74]. Previous experimental studies have suggested that overexpression of α-synuclein would reduce the release of neurotransmitter by inhibiting the reclustering of synaptic vesicles after endocytosis [75]. Such biological process would produce a physiological defect in synaptic vesicle recycling before detectable neuropathology. Besides, most cases of LBD are often accompanied by varying degrees of AD pathology, including neurofibrillary tangles (NFTs) and senile plaques [76]. Tau is the major structural component of NFTs that intraneuronal aggregates of hyperphosphorylated and misfolded tau that become extraneuronal when tangle-bearing neurons die, which would contribute to cognitive impairment [77–79]. Previous studies have also suggested the coexistence of tauopathies and synucleinopathies in LBD [80]. In addition, neocortical α-synuclein, tau, and amyloid pathologies can co-occur at the advanced stage of LBD, suggesting a potential synergistic interaction of these pathologies [81]. Specifically, experimental studies in animal and cell model systems have shown that pathogenic species of synuclein fibrils can facilitate the trans-synaptic spread of both tauopathy and synucleinopathy with strain-like properties, which would aggravate the severity and progression of LBD [81]. A recent systematic review also indicated that compared to people with pure dementia with Lewy bodies, those with mixed Lewy body and AD neuropathologies suffered more severe cognitive impairment before death [82]. The underlying co-pathology of these common neurodegenerative diseases suggested the potential value of simultaneous prevention and treatment of these diseases.

Our study is not without limitations. First, we only focused on European ancestry due to the current large-scale GWASs of LBD, AD, and PD were only available for the European population. Further investigation is needed to explore the genetic architecture of LBD in other populations. Second, the genetic associations of rare variants were unable to be evaluated since SNPs with MAF less than 0.01 were automatically filtered in MTAG analysis.

## Conclusions

In summary, our findings provide strong evidence of genetic correlations between LBD and AD as well as LBD and PD. We identified novel LBD-associated genetic loci as well as novel LBD-associated genes. We also highlighted the critical role of neurofibrillary tangles in the development of LBD. More importantly, our findings not only advance the understanding of genetic determinants of LBD but also provide novel insights into the shared genetic etiology of LBD, AD, and PD from functional and biological pathway levels. Shared common biological mechanisms could provide novel insight to simultaneously prevent and treat these diseases.

## Supplementary Information

**Additional file 1: Table S1**. Summary of GWAS data. **Table S2**. Single trait LDSC of LBD, AD, and PD GWAS. **Table S3**. Genome-wide significant SNPs (*P*_mtag_ < 5×10^-8^) of MTAG results of LBD and functional annotations. Association statistics of these SNPs from LBD GWAS and one-sided and two-sided ASSET analysis were also provided. **Table S4**. Genomic risk loci identified by FUMA using LBD GWAS. **Table S5**. Lead SNPs (*P* < 5×10^-8^ and r^2^ < 0.1) of LBD GWAS and MTAG results of LBD. **Table S6**. Summary of annotations of 2,160 validated SNPs. **Table S7**. GCTA-COJO analysis results. **Table S8**. 90% credible sets of 13 genomic risk loci. **Table S9**. PP4 of SNPs included in colocalization analysis. **Table S10**. Functional enrichment analysis using GARFIELD. **Table S11**. Gene-based analysis for MTAG results of LBD (after Bonferroni correction). **Table S12**. Transcriptome-wide association study using joint tissue imputation models and MTAG results of LBD (after Bonferroni correction in each tissue). **Table S13**. Candidate genes identified by both GCTA-fastBAT and TWAS. **Table S14**. Enrichment analysis for 40 candidate genes associated with LBD.

**Additional file 2: Figs. S1-S13**. Regional association plots of 13 genomic risk loci associated with LBD identified from MTAG.

## Data Availability

The datasets generated and/or analyzed during the current study are publicly available. GWAS summary data for LBD is available from GWAS Catalog (study accession: GCST90001390) at https://www.ebi.ac.uk/gwas/ [83], for AD is available from the Complex Trait Genetics lab at https://ctg.cncr.nl/software/summary_statistics/ [84], and for PD is available from the MRC IEU OpenGWAS database (GWAS ID: ieu-b-7) at https://gwas.mrcieu.ac.uk/datasets/ [85]. Data used in LDSC analysis can be obtained at https://alkesgroup.broadinstitute.org/LDSCORE/ [31]. JTI models of gene expression in 13 brain tissues are available from Zenodo at 10.5281/zenodo.3842289 [86].

## Abbreviations

AD: Alzheimer’s disease
ASSET: Association analysis based on subsets
CADD: Combined annotation-dependent depletion
fastBAT: Fast set-based association analysis
FUMA: Functional mapping and annotation of genetic associations
GARFIELD: GWAS analysis of regulatory or functional information enrichment with LD correction
GTEx: Genotype-Tissue Expression
GWAS: Genome-wide association study
JTI: Joint-tissue imputation
LBD: Lewy body dementia
LD: Linkage disequilibrium
LDSC: Linkage disequilibrium score regression
MAF: Minor allele frequency
maxFDR: Maximum false discovery rate
MHC: Major histocompatibility complex
MTAG: Multi-trait analysis of GWAS
OR: Odds ratios
PD: Parkinson’s disease
PP: Posterior probability
TWAS: Transcriptome-wide association analysis
