# Supplementary material for: Pinpointing novel risk loci for Lewy body dementia and the shared genetic etiology with Alzheimer’s disease and Parkinson’s disease: a large-scale multi-trait association analysis
Source: BMC Med. 2022 Jun 22;20:214. doi: 10.1186/s12916-022-02404-2 (PMC9214990; doi:10.1186/s12916-022-02404-2)
Supplement: Supplementary file 2 — Additional file 2: Figs. S1-S13. Regional association plots of 13 genomic risk loci associated with LBD identified from MTAG. [file 12916_2022_2404_MOESM2_ESM.pdf]

Figs. S1-S13. Regional association plots of 13 genomic risk loci.

The index SNP, which is the SNP with the smallest *p*-value in the genomic risk loci defined by FUMA, was indicated by a purple circle. The association of an individual variant is plotted as -log10 (*P* value) against chromosomal position. The y axis shows the recombination rate estimated from 1000 Genomes Project EUR data. For each locus, from top to the bottom, the LocusZoom plot was derived from summary statistics of $MTAG_{LBD}$, $GWAS_{LBD}$, $GWAS_{AD}$, and $GWAS_{PD}$, respectively, with the genes located in this region presented.

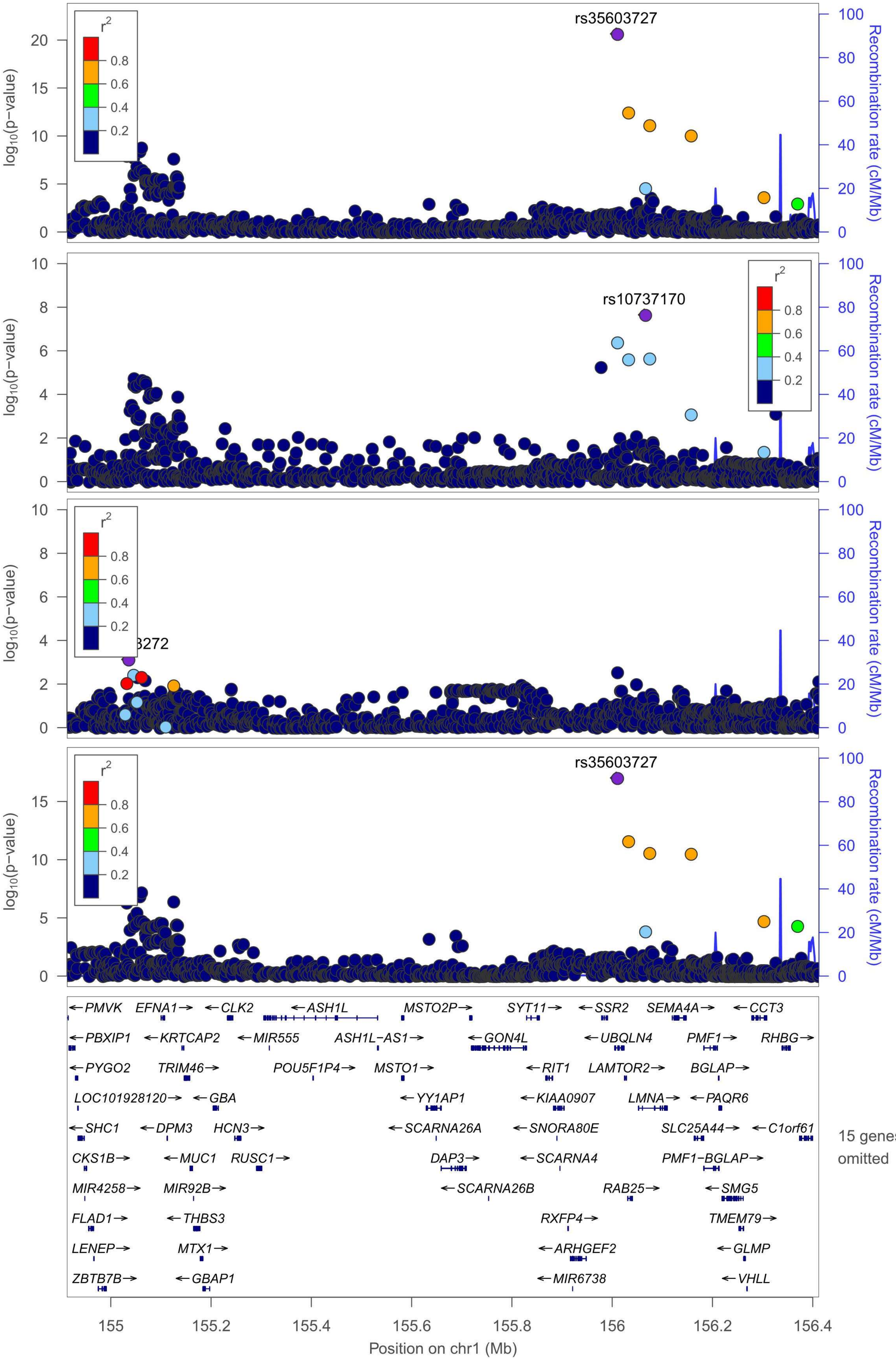

Fig. S1: The LocusZoom plot of genomic risk loci 1q22

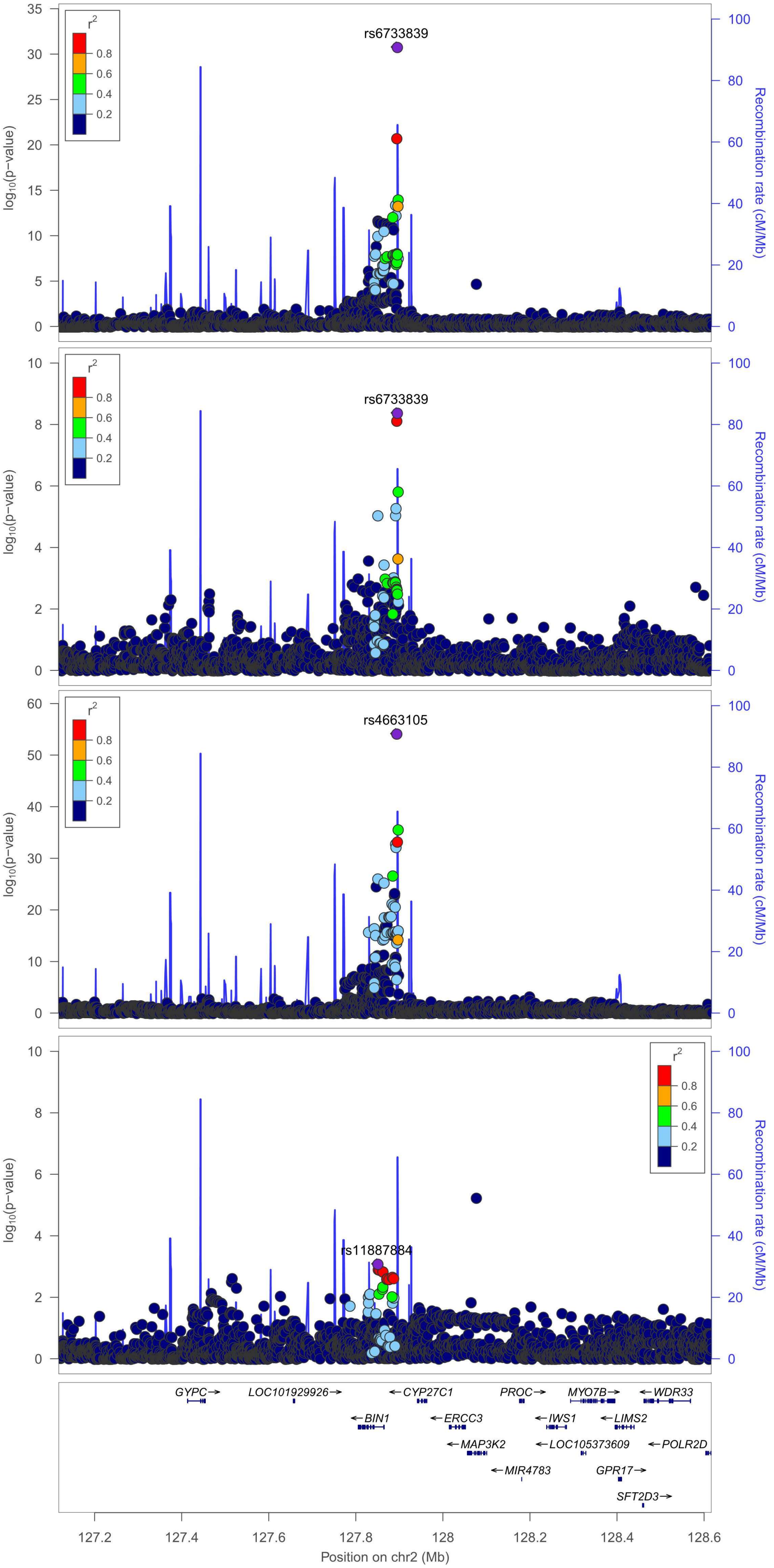

Fig. S2: The LocusZoom plot of genomic risk loci 2q14.3

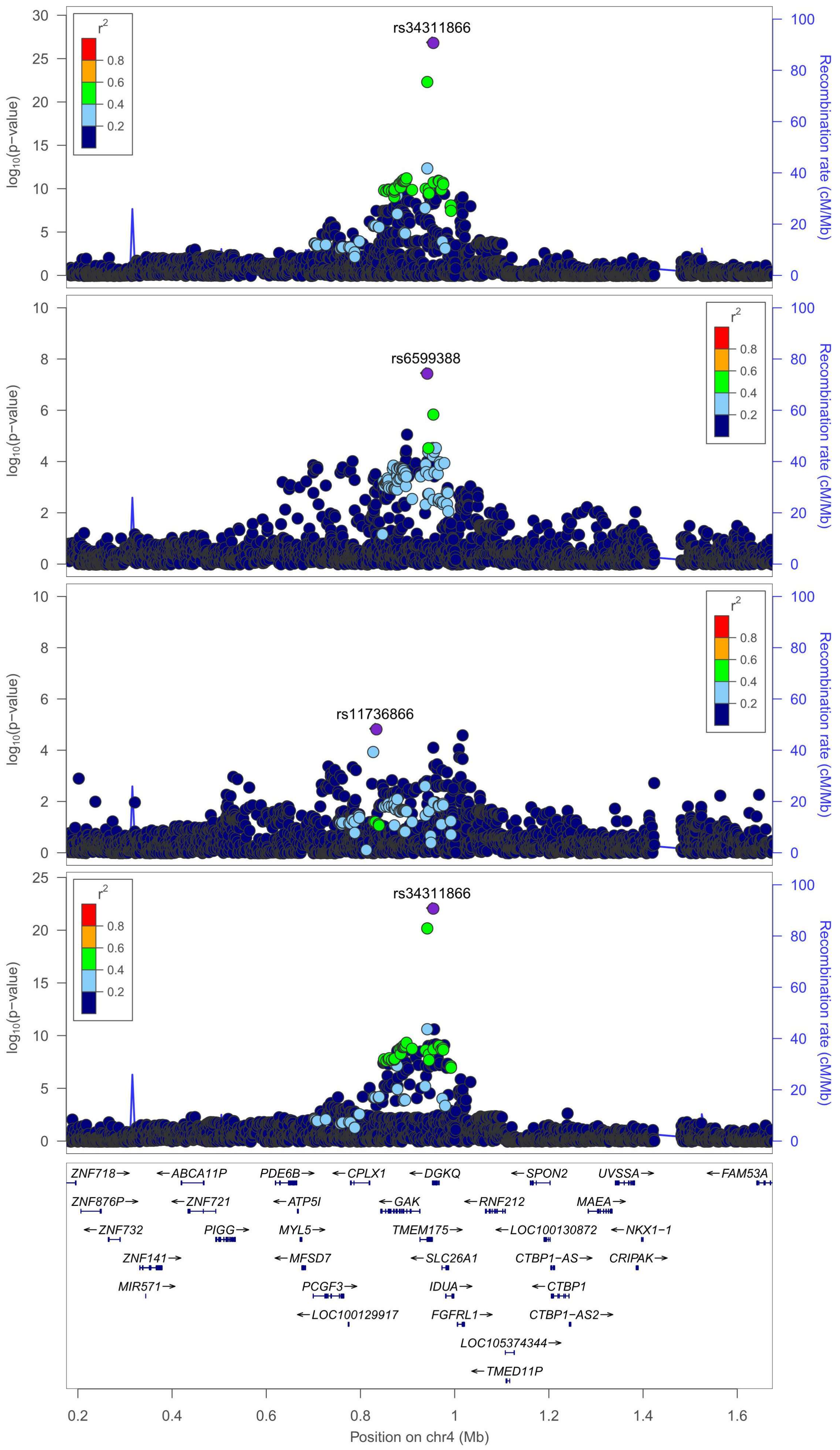

Fig. S3: The LocusZoom plot of genomic risk loci 4p16.3

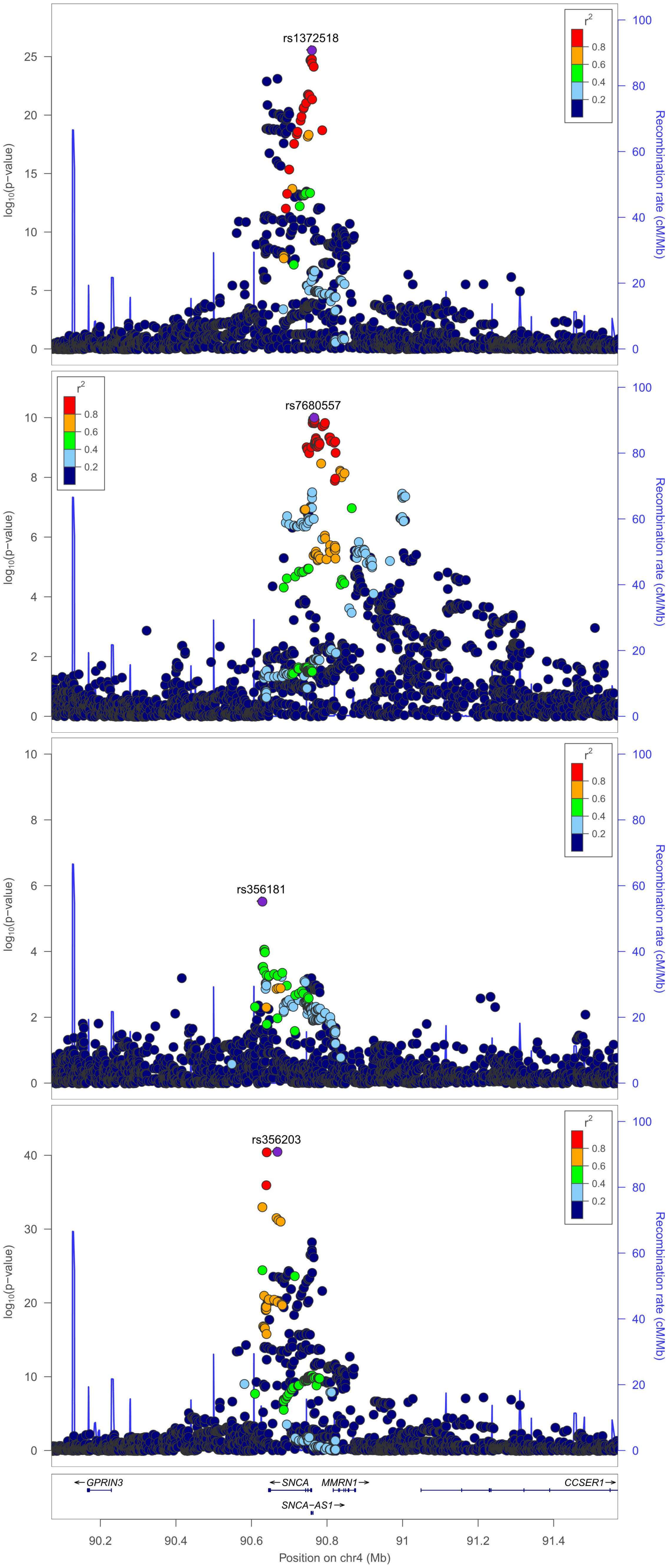

Fig. S4: The LocusZoom plot of genomic risk loci 4q22.1

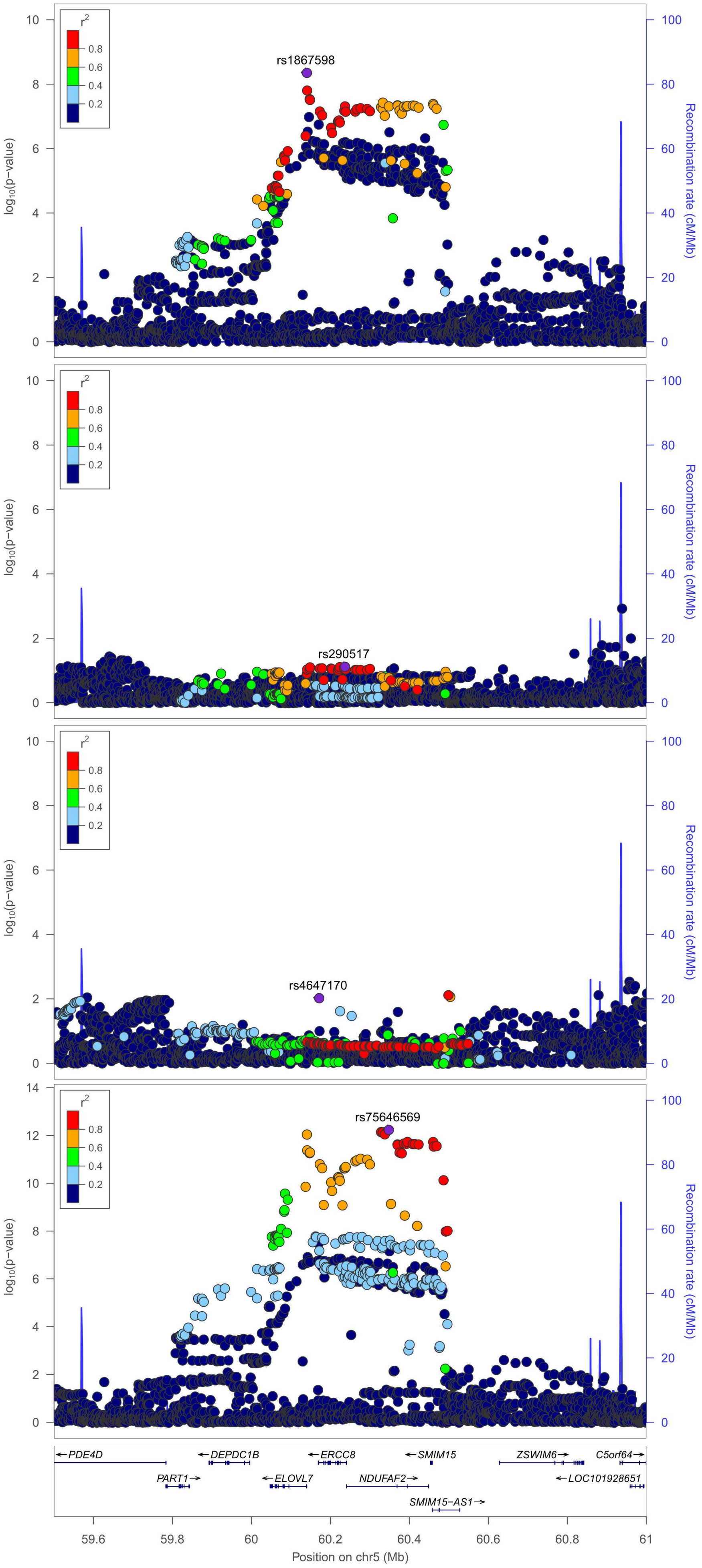

Fig. S5: The LocusZoom plot of genomic risk loci 5q12.1

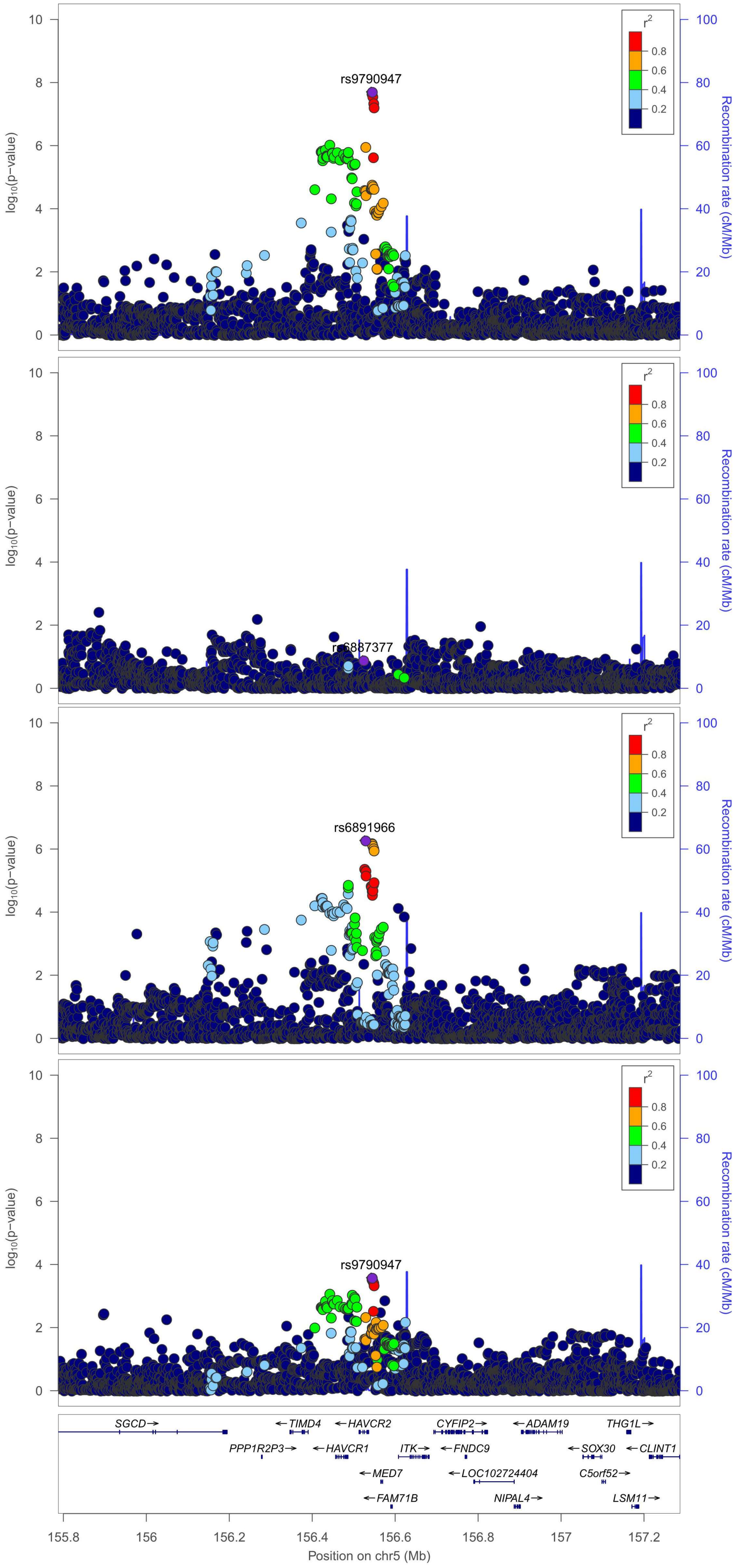

Fig. S6: The LocusZoom plot of genomic risk loci 5q33.3

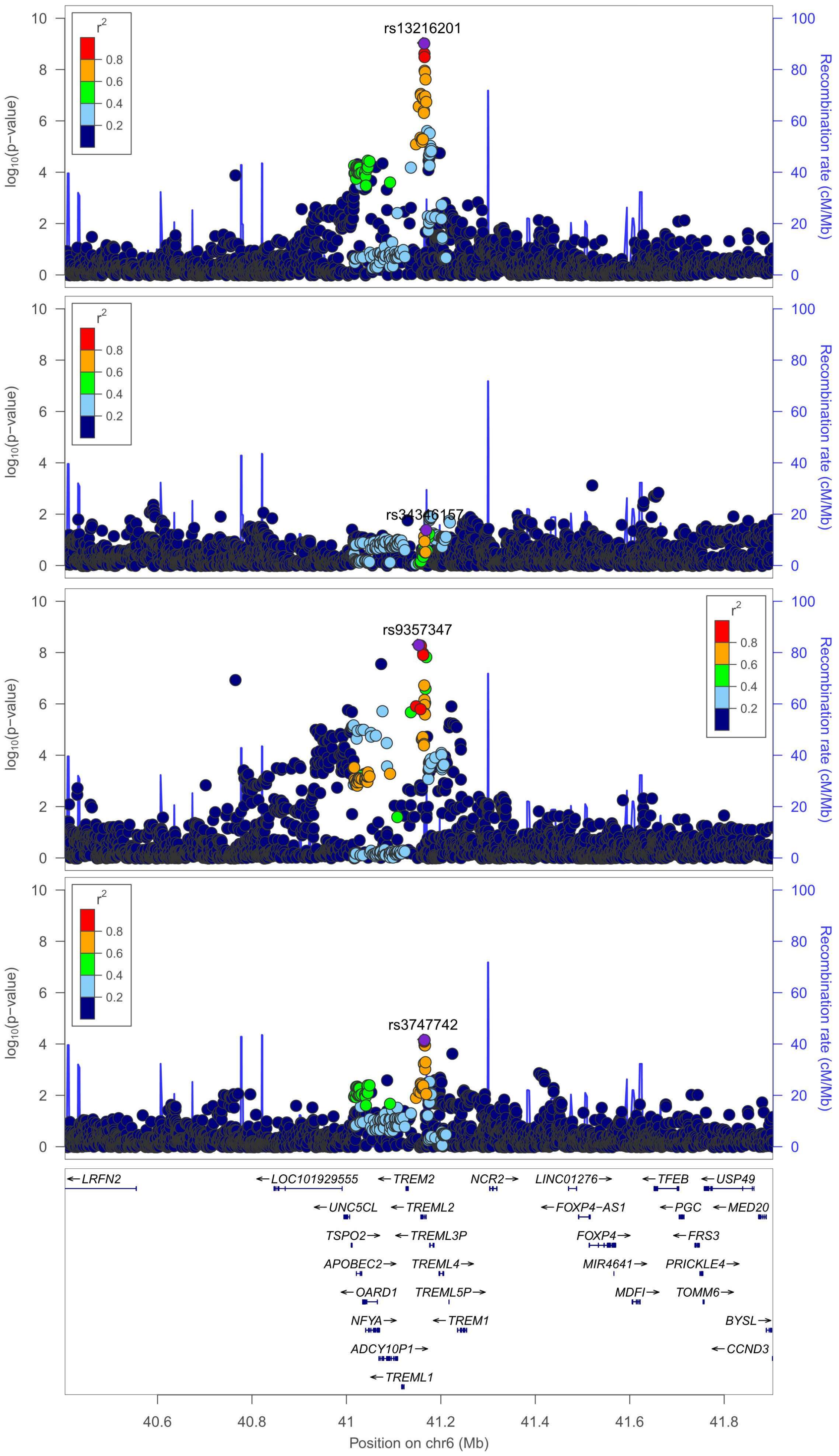

Fig. S7: The LocusZoom plot of genomic risk loci 6p21.1

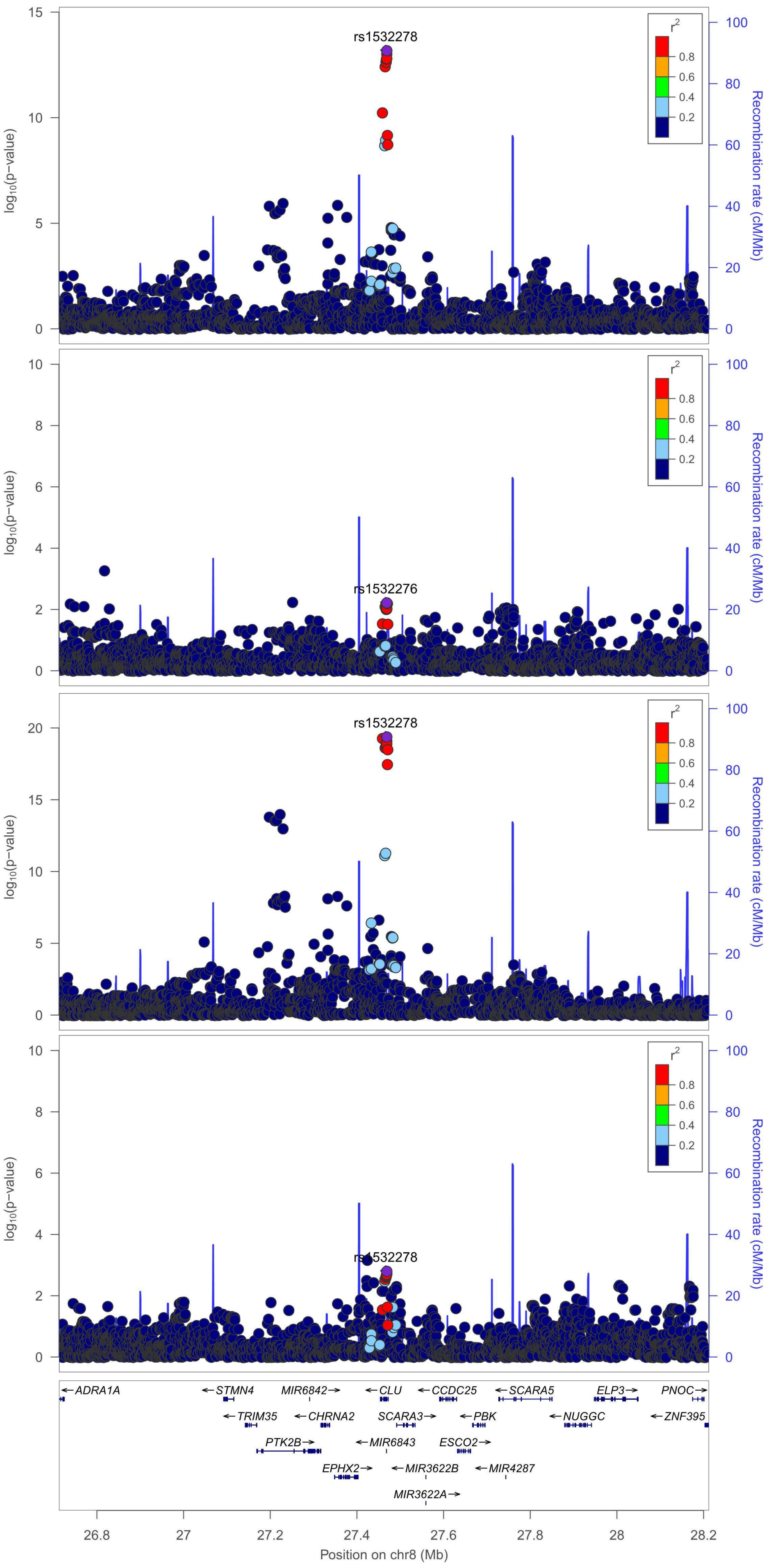

Fig. S8: The LocusZoom plot of genomic risk loci 8p21.1

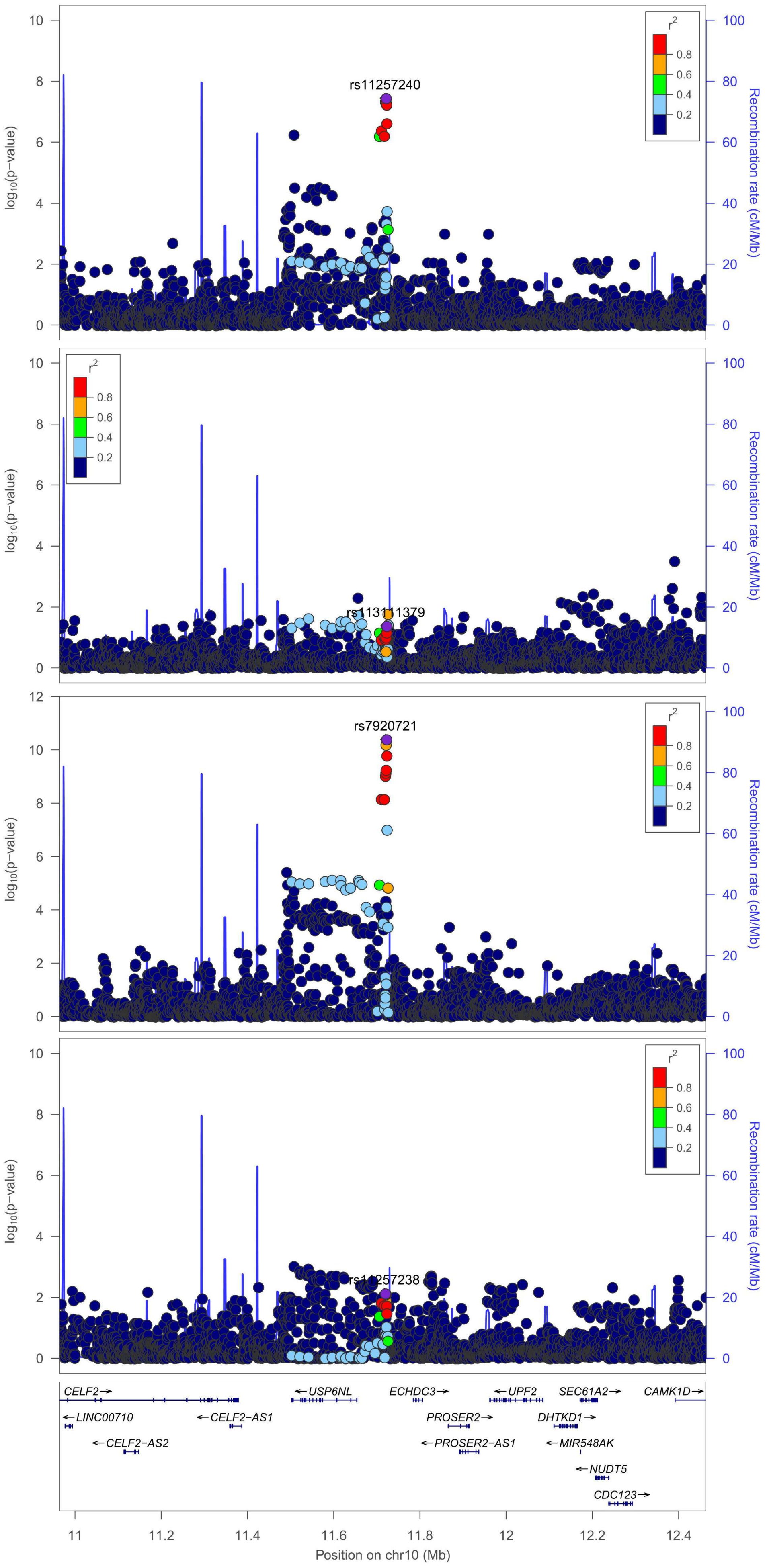

Fig. S9: The LocusZoom plot of genomic risk loci 10p14

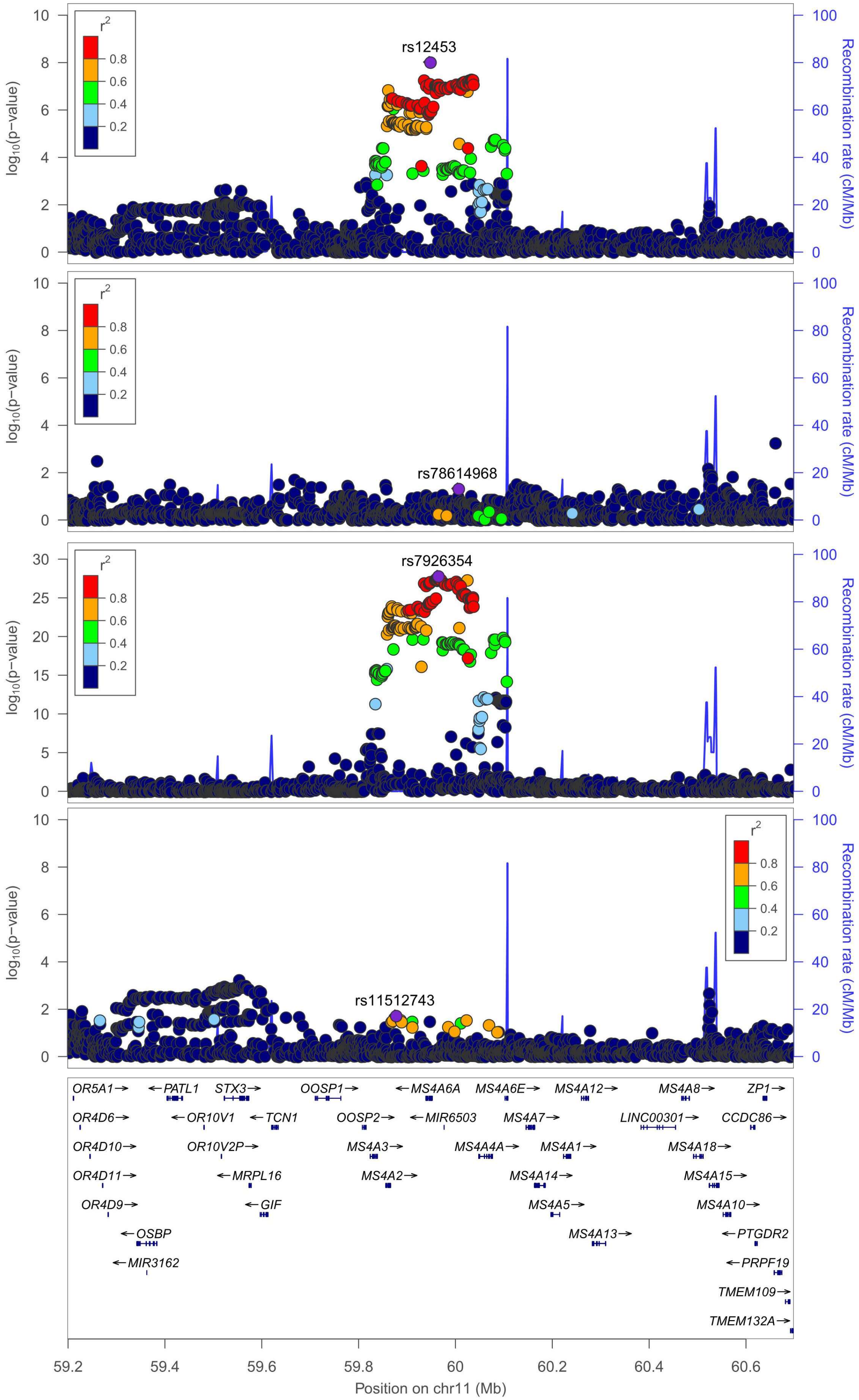

Fig. S10: The LocusZoom plot of genomic risk loci 11q12.2

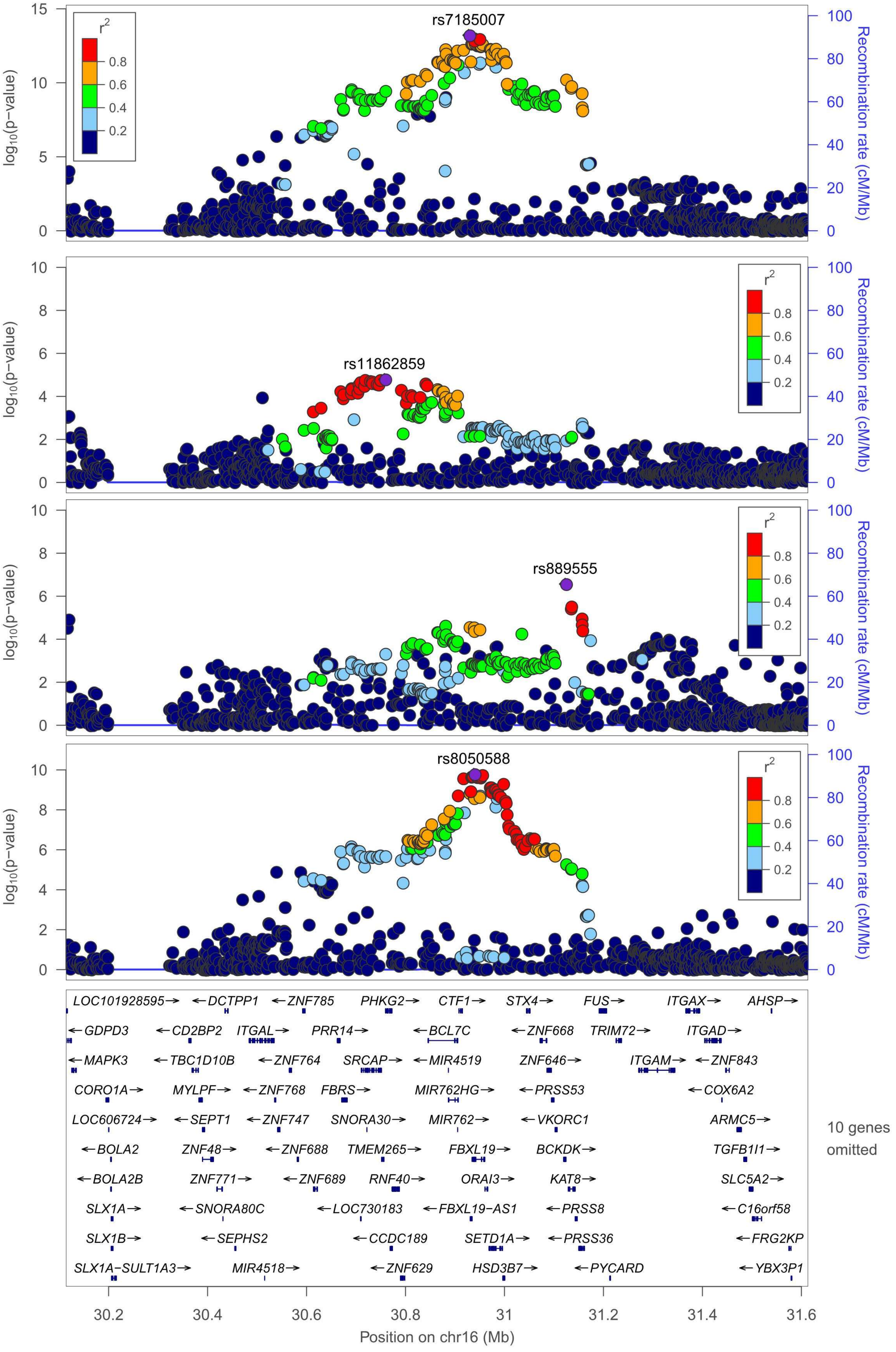

Fig. S11: The LocusZoom plot of genomic risk loci 16p11.2

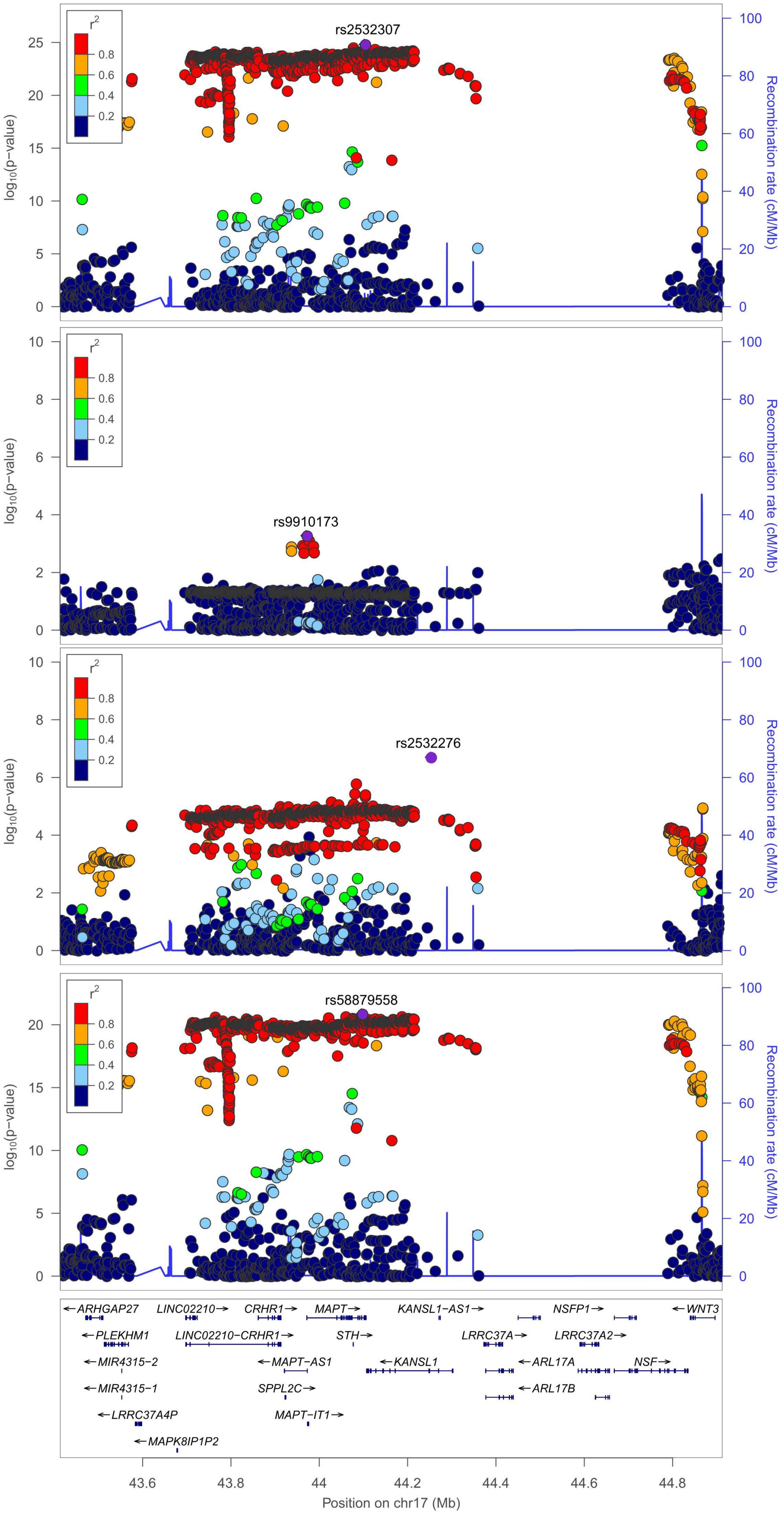

Fig. S12: The LocusZoom plot of genomic risk loci 17q21.31

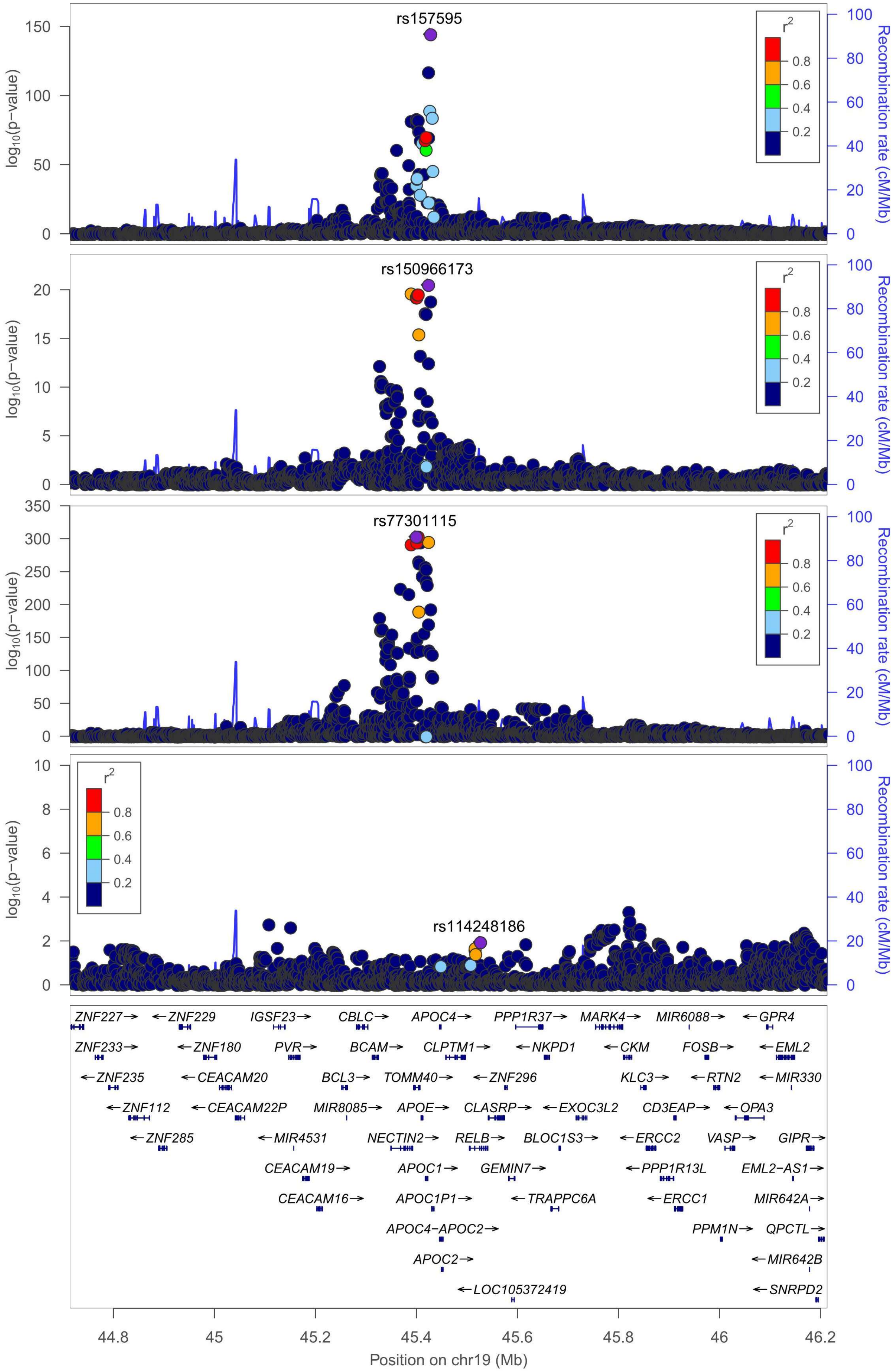

Fig. S13: The LocusZoom plot of genomic risk loci 19q13.32
